# Non‐Random Mortality in an Experimental Oyster Restoration

**DOI:** 10.1111/eva.70128

**Published:** 2025-07-06

**Authors:** Sarit Truskey, Erik Sotka, Jonathan Grabowski, Nicole M. Kollars‐Kjersten, Katie E. Lotterhos, Eric Schneider, A. Randall Hughes

**Affiliations:** ^1^ Marine Science Center Northeastern University Nahant Massachusetts USA; ^2^ Department of Biology College of Charleston Charleston South Carolina USA; ^3^ Division of Marine Fisheries Rhode Island Department of Environmental Management Jamestown Rhode Island USA

**Keywords:** eco‐evolutionary dynamics, evolution, experimental restoration, habitat restoration, oyster, sourcing practice

## Abstract

Ecological restoration has emerged as a prominent conservation and management strategy widely touted for its utility in evaluating ecological theories when designed experimentally. In comparison, restoration has been underutilized to investigate evolution‐oriented questions, despite the importance of evolutionary processes in conservation and management settings. Here, we leverage an experimental restoration approach using the eastern oyster, *Crassostrea virginica* , an economically valuable and ecologically important reef‐building foundation species. Previous small‐scale manipulations of oyster source identity highlight the potential evolutionary implications of sources used in restoration, yet have rarely been empirically evaluated at the scale of a restored reef. We sourced juvenile oysters from four commercial hatcheries spanning a broad geographic range along the Atlantic coast of the United States to build restored oyster reefs of diverse initial source composition in a single New England estuary. We characterized four distinct genetic clusters associated with hatchery source using SNP genotyping data and examined whether the frequencies of these genetic clusters on our mixed reefs shifted over the course of our restoration experiment. We documented strong shifts in the relative abundance of certain genetic lineages, consistent with differential mortality among oyster sources. Further, we found significant variation in ecologically relevant traits, including multi‐parasite infection patterns and oyster size, associated with source identity. Oyster condition index, a commonly used proxy for oyster health, was associated with higher relative mortality over time. Our research highlights how evolutionary processes can influence restoration demographics and how, concurrently, restoration can serve as a powerful platform for gaining fundamental, and sometimes unexpected, insights into eco‐evolutionary dynamics.

## 1 Introduction

Understanding the maintenance of genetic and phenotypic variation in a range of environmental contexts, and its consequences for the fitness of populations, is of central interest in evolutionary biology (Hilbish and Koehn 1987; Lewontin 1990; Lande and Shannon 1996). However, the ability to evaluate such links is often challenging or impractical in natural to semi‐natural settings where within‐species variation in genetics and traits are often spatially confounded with environmental differences. Management and conservation interventions, through the manipulation of within‐species variation and/or environmental differences and the monitoring of program outcomes, have helped to address this gap and to inform our understanding of evolution. For instance, forestry provenance trials, where the growth and survival of seeds of different geographic origin planted at various test sites are examined over time, have informed management goals for delineating seed transfer zones, while also affording critical insight into our basic understanding of local adaptation (Aitken and Bemmels 2016). In addition, temporal sampling within fish stocking and supplementation programs, particularly for salmonids, has provided evidence for human‐mediated contemporary evolution of managed populations, along with ready examples of maladaptation and strong domestication selection associated with captive (hatchery) rearing (Araki et al. 2008; Christie et al. 2012). Management interventions have also provided some of the seminal examples of eco‐evolutionary dynamics in real‐world settings (e.g., Post et al. 2008; Palkovacs and Post 2009).

Ecological restoration is an increasingly common conservation and management tool (Suding 2011), with commitments to expand restored acreage moving forward (e.g., United Nations Global Decade on Ecosystem Restoration). The potential for ecological restoration to be used to validate ecological theories has been widely emphasized (Bradshaw 1987; Young et al. 2005; Perring et al. 2015), and notably implemented, for instance, in testing predictions about community assembly and species diversity (e.g., Baer et al. 2005), as well as ecosystem function (e.g., Camill et al. 2004; Tilman et al. 2006). In comparison, ecological restoration has been an underutilized approach for testing evolution‐oriented questions (Wainwright et al. 2018), despite the recognized importance of evolution in other management settings (e.g., selective breeding; conservation genetics) and the clear relevance to restoration practice and outcomes (Stockwell et al. 2016; LaRue et al. 2017; Hendry et al. 2024). For example, in many settings, restoration requires the active addition or supplementation of target species, whether from natural populations or commercially produced stocks (e.g., salmonids, Cuenco et al. 1993; corals, Shafir et al. 2006). Decisions about how many and which sources to use offer a unique opportunity to explore relationships between intraspecific variation and selection in a management‐relevant context, often with important consequences for whether restoration is successful. However, the evolutionary implications of such sourcing decisions and their implications for restoration success have rarely been empirically tested.

Despite few published studies that have actively integrated questions about evolution into an experimental restoration approach, the relevance and promise of such integration are evident. Designed experimentally, restorations can be used as “common gardens” to examine interactions between the geographic source of populations and their performance in a given environment (e.g., Gellie et al. 2016; Bucharova et al. 2017; Wood et al. 2020; Young et al. 2020; Bailey et al. 2021). Restoration experiments can also provide a valuable setting in which to examine the presence and consistency of phenotypic selection and contemporary evolution (e.g., Bailey and Kinnison 2010; Kulpa and Leger 2013; Magnoli and Lau 2020). Akin to studies of selection within novel colonizing populations during species range shifts or introductions (e.g., Moran and Alexander 2014), evolutionary‐focused restoration experiments have the added bonus of more precise information pertaining to the source populations used and the ability to control for potentially confounding influences, like initial population densities and time since establishment. Experimental ecological restoration thus presents an as‐of‐yet underutilized opportunity to concurrently improve decisions around restoration practices and to gain basic insight into eco‐evolutionary dynamics at larger scales and in a range of environmental contexts (Stockwell et al. 2016; Hendry et al. 2024; Nadeau et al. 2024). This approach, while broadly valuable, is likely to be particularly relevant for (i) habitat‐providing or foundation species, which are often the target of restoration programs and through which variation in genetics and traits may be expected to exert strong ecological consequences (Hendry 2017), and (ii) programs dependent on supplementation and ex‐situ propagation (e.g., in nursery or hatchery), where managers directly select the sources of genetic material used in restoration.

The eastern oyster *Crassostrea virginica* is an economically valuable and ecologically important reef‐building foundation species distributed along the Atlantic and Gulf coasts of North America, and it is one of the most frequently and extensively restored marine species (Grabowski et al. 2012; Bersoza Hernández et al. 2018). In addition to its utility for testing ecological predictions relating to tidal zonation (Fodrie et al. 2014) and habitat setting (Grabowski et al. 2005), experimental oyster restoration provides a unique opportunity to answer questions about rapid evolutionary change. Oyster reefs occur as discrete patches of many individuals, providing a means to measure and manipulate reef genetic identity and diversity through the use of different oyster sources and source combinations (e.g., Hanley et al. 2023). Such methods are of direct relevance to restoration practice. Reef restoration occurs through either (i) the deployment of hard substrate on which larvae can settle and reefs can accrue, (ii) the transplantation of hatchery‐produced juvenile or adult oysters to “seed” reefs, or (iii) a combination of the two. In regions characterized by low adult densities and limited natural recruitment, such as the northeastern United States (Grizzle and Ward 2016), hatchery production and transplantation are currently an indispensable part of oyster restoration programs, imparting practical importance to questions of source selection and diversity. While hatchery‐supported supplementation‐based oyster reef restoration programs have been ongoing in various regions for decades, it remains unclear how sourcing decisions regarding the genetic material used in restoration translate to reef‐level ecological and evolutionary dynamics (but see Hanley et al. 2023).

Small‐scale manipulations of oyster source identity highlight the probable restoration‐relevance of sourcing decisions, with evidence for variation in juvenile oyster traits (Hughes et al. 2019) and some examples of local adaptation (Burford et al. 2014; Olympia oyster *Ostrea edulis* ; Bible and Sanford 2016; but see Hughes et al. 2017). Commercial oyster breeding programs also demonstrate the potential for genetic variation in a variety of traits, including resistance to and tolerance of individual parasites (Ben‐Horin et al. 2018; Ford and Haskin 1987; Proestou et al. 2019), growth rate (Evans and Langdon 2006; Rawson and Feindel 2012), low‐salinity survival (McCarty et al. 2020), and more. Yet, genetic variation for traits among selectively bred commercial lines is generally a poor predictor of performance in the field (e.g., Rawson and Feindel 2012; Proestou et al. 2016) and of persistence over multiple reproductive generations (Carlsson et al. 2008; Hare et al. 2006).

Here, we conducted a multi‐year field experiment in which we sourced juvenile oysters from four commercial hatcheries (i.e., sources) across a broad geographic range, and we used these oysters to build restored oyster reefs of diverse initial source composition. We characterized four distinct genetic clusters associated with hatchery source using RAD‐seq generated SNP genotyping data and examined whether the frequencies of these genetic clusters on our mixed‐cluster reefs shifted over a time period defined by high oyster mortality. If survival at the restoration site is mediated by the source identity of oysters and varies among sources, we would expect consistent shifts in the relative frequencies of genetic clusters on reefs over time, reflecting patterns of differential mortality (selection), as opposed to random fluctuations in reef‐level source frequencies. Variation in survival among oyster sources could reflect underlying differences in adaptation to spatially variable selection pressures coincident with the regional locations of the source hatcheries (e.g., to well‐known drivers of selection in marine invertebrates, like temperature, salinity, or parasite exposure; Sanford and Kelly 2011). Oysters from the source hatcheries most local to the restoration site, in this case, may be expected to show higher performance. Alternatively, differences in survival may also reflect hatchery‐induced genetic factors, such as reduced genetic diversity or elevated inbreeding, which may influence survival outcomes following transplantation (e.g., salmonids, Araki et al. 2008). Altogether, we addressed the following questions: (1) Are there non‐random shifts in the oyster reef‐level frequencies of genetic clusters over the course of restoration? (2) Is there significant variation associated with genetic cluster identity in a suite of ecologically relevant oyster traits? (3) Which, if any, initial oyster traits are associated with shifts in genetic cluster frequencies over time on the experimental reefs? (4) Do oysters from the different genetic clusters vary in initial levels of genetic diversity and in how genetic diversity within each group changes over the course of the restoration?

## 2 Materials and Methods

### 2.1 Eastern Oyster Restoration in Rhode Island

While historically abundant in southeast New England coastal waters, wild eastern oyster populations have declined to less than 1% of these abundances (Beck et al. 2011; Zu Ermgassen et al. 2012). Remnant natural oyster populations currently persist in fragmented subtidal habitats within Rhode Island's South Shore coastal ponds and Narragansett Bay. The Rhode Island coastal system has also become a focal area for oyster aquaculture and restoration, with efforts ongoing across the region for several decades (Griffin 2016). Given low rates of natural recruitment, restoration in this system is entirely reliant on reef supplementation supported via hatchery propagation of juveniles and has often incorporated commercial aquaculture lines given challenges associated with sourcing local broodstock (Griffin 2016).

### 2.2 Restoration Experiment and Sample Collection

**FIGURE 1:**
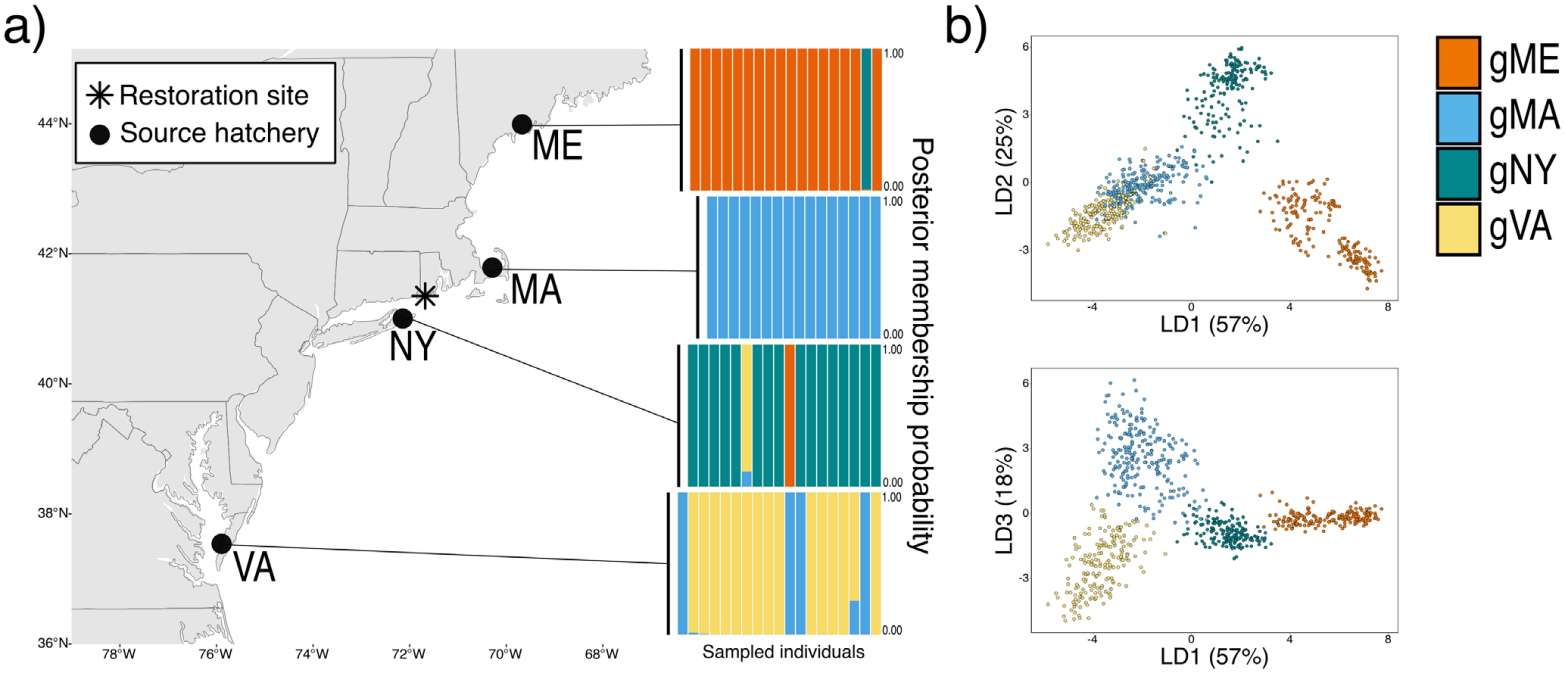
(a) Approximate geographic locations of the four commercial hatchery sources for the juvenile oysters used to build experimental oyster reefs. Genetic assignments via DAPC of individuals from these initial source hatchery samples to the four best genetic clusters (gME, gMA, gNY, and gVA) are provided in the composition plots on the right‐hand side of the map. (b) Scatterplots showing relationships among the best four genetic clusters identified in DAPC with the first linear discriminant on the *x*‐axis and second and third linear discriminants on the *y*‐axis for the top and bottom plots, respectively. Orange = gME; blue = gMA; green = gNY; yellow = gVA.

In April 2017, we collaborated with local oyster farmers to source oyster eyed‐larvae from four commercial hatcheries for the construction of new experimental oyster reefs in Ninigret Pond (Charleston, Rhode Island, USA). Hatchery sources were selected across a broad geographic sampling range to maximize diversity (Figure 1a). They included two regional hatcheries, one from Massachusetts (MA) and one from New York (NY), and two more distant hatcheries, one from Maine (ME) and one from Virginia (VA). Of these hatcheries, two (MA and VA) had a history of use in state‐directed oyster reef restoration programs in Rhode Island.

Through a partnership supported by the USDA Natural Resource Conservation Service's Environmental Quality Incentives Program (EQIP) Oyster Reef Restoration Initiative, a local Rhode Island hatchery facility received oyster eyed‐larvae from each source hatchery, set the eyed‐larvae on dead oyster and/or clam shell (hereafter referred to as spat‐on‐shell oysters), and distributed the spat‐on‐shell oysters to three local oyster growers (Grower 1: MA; Grower 2: ME, NY, VA; Grower 3: NY, VA) who maintained these juvenile oysters on separate leased oyster farm plots prior to reef construction. Immediately prior to reef construction, we collected 20 juvenile spat‐on‐shell oysters from each grower‐hatchery source combination for genetic analysis (*n* = 6 combinations; Grower 1‐MA; Grower 2‐ME; Grower 2‐NY; Grower 2‐VA; Grower 3‐NY; Grower 3‐VA).

**FIGURE 2:**
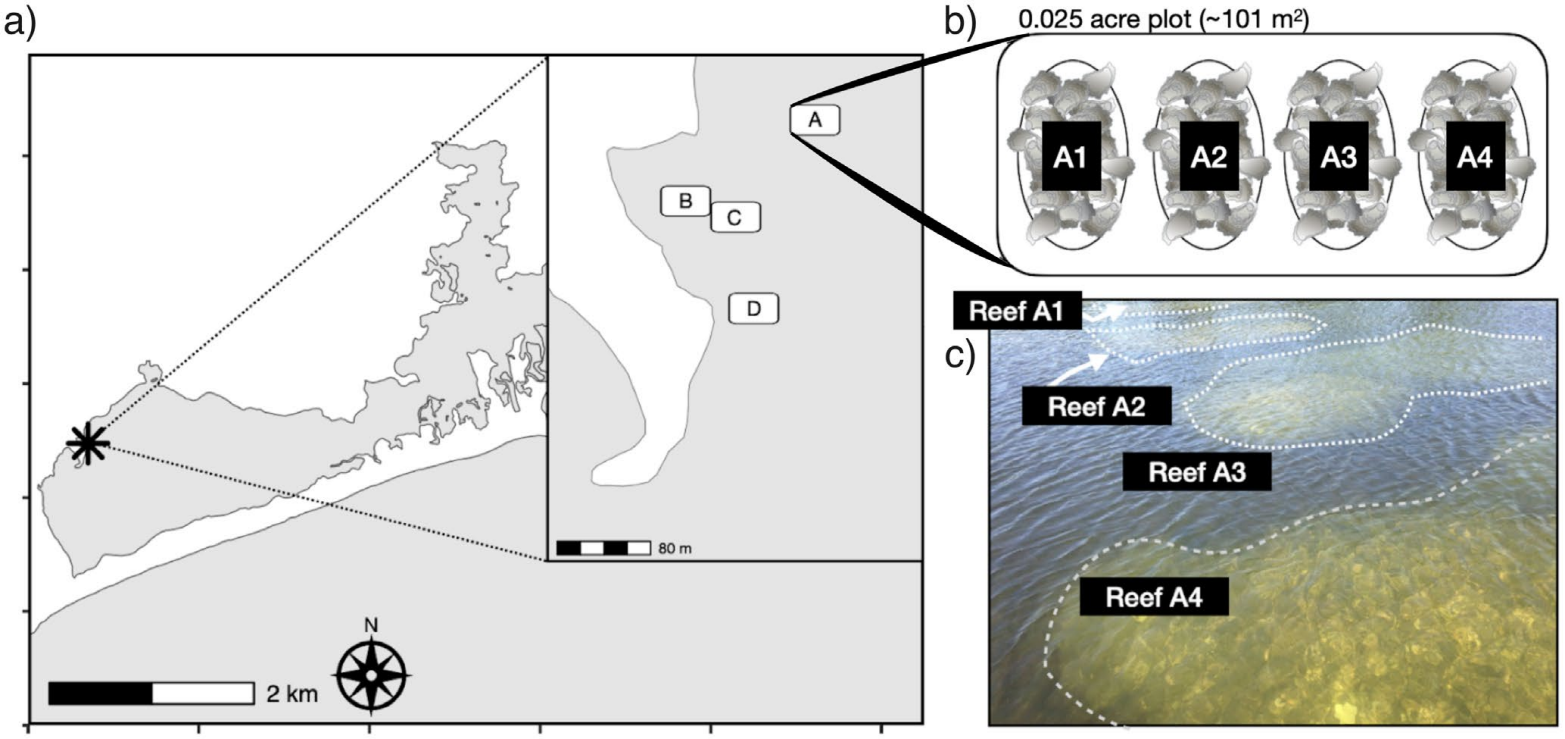
(a) Map of experimental restoration location in Ninigret Pond, RI, showing the location of experimental blocks A–D. (b) Schematic of the layout of individual restored reefs in each experimental block, with (c) a reference photograph of what this arrangement of reefs looked like in the field.

In October and November 2017, we constructed 16 subtidal reefs within a no‐harvest Shellfish Management Area in Ninigret Pond. Reefs were created across four 0.025‐acre experimental blocks, with four reefs per block (Figure 2). Each reef consisted of a base layer of 0.25 cubic yards of dead oyster or clam shell deployed in October and topped with 1.25 cubic yards of spat‐on‐shell oysters in early November from the stock grown out by the local oyster growers. Our experiment was originally designed to seed three reefs per block with a different single hatchery source from the four available hatchery sources and to seed the fourth reef per block with a mixed combination of the three sources (e.g., Reef 1: ME, Reef 2: MA, Reef 3: NY, Reef 4: ME+MA+NY). However, analysis of the hatchery samples from oyster growers collected prior to reef construction revealed an early, unintended mixing of some of the sources (Appendix S1; Figure S1), resulting in mixtures of multiple sources on 12 out of the 16 constructed reefs. Thus, we focused our analyses on the actual genetic composition of each reef as determined by genetic sampling at two time points (fall 2018, fall 2020).

In fall 2018, we haphazardly sampled live oysters from each reef on scuba or snorkel (*N* = 512 individuals total, 32 per reef). Oysters were put on ice and transported to the Northeastern University Marine Science Center where they were held at −80°C until DNA extraction. By the fall of 2020, live oyster densities on all experimental reefs had declined, reflecting mortality of the original planted oysters and a lack of recruitment, consistent with other data from this system (Barrett et al. 2024). To assess whether this mortality was associated with a consistent change in the genetic composition of surviving oysters on reefs, we repeated our sampling in fall 2020 and compared the resulting reef genetic profiles to those from fall 2018. Reef sample sizes varied at this time point due to low live abundances (*N* = 249 individuals total, ranging from 8 to 32 per reef).

### 2.3 DNA Extraction, RADseq Library Preparation, and Bioinformatics

Genomic DNA was extracted using the E‐Z 96 Tissue DNA Kit (Omega‐Biotek, Norcross, GA) following the protocol for animal tissue with tissue centrifugation. Double digest restriction‐site‐associated DNA (ddRAD) libraries with individually barcoded samples were then prepared in three batches following protocols in Parchman et al. (2012). The three batches that were separately prepared and sequenced were: (1) initial hatchery samples (*n* = 120; referred to as the initial batch), (2) individuals sampled from reefs in fall 2018 (*n* = 480; 30 oysters/reef for 16 reefs; referred to as the fall 2018 batch), and (3) individuals sampled from reefs in fall 2020 (*n* = 248; referred to as the fall 2020 batch). An additional subset of oysters sampled in 2019 and 2021 were included in the fall 2020 batch (*n* = 37 oysters) to confirm an apparent missampling of one reef in fall 2018, but removed from downstream analyses. During library preparation, we randomly assigned samples from each reef to plate positions and checked for sufficient interspersion to minimize batch effects related to reef identity. For additional details on ddRAD library preparation, see Appendix S2. All libraries were sequenced with 100‐bp single‐end sequencing on an Illumina platform. The initial and fall 2020 batches were each separately sequenced on a single lane on the Illumina HiSeq 2500 platform at Tufts University Core Facility Genomics (Boston, MA) and the larger fall 2018 batch was sequenced on two lanes on the Illumina NovaSeq 6000 platform at the University of Texas at Austin Genomic Sequencing and Analysis Facility (Austin, TX).

### 2.4 SNP Calling, Filtering, and Defining Datasets

We used a conda v22.11.1 (Anaconda Software Distribution 2020) environment to manage bioinformatic packages for this study on the Northeastern University High‐Performance Computing Cluster. For each library, read quality was evaluated using FastQC v0.11.9 (S. Andrews 2010). Raw sequence files were then demultiplexed using the process_radtags function in STACKS v.2.41 (Catchen et al. 2013) under default parameters with the option to rescue barcodes with up to one mismatch. Demultiplexed individual fastq files were then aggregated in a single combined library folder and processed together in the following steps. Read trimming and mapping, followed by SNP calling and genotyping, were performed with the *dDocen* *t* pipeline v2.9.4 (Puritz et al. 2014). Default settings within *dDocent* were used if not stated otherwise. Trimmed reads were mapped to the *C. virginica* genome pruned for haplotigs (Puritz et al. 2024) with the match score, mismatch score, and gap penalty set to 1,3, and, 5, respectively, as previously used with ddRAD oyster data (Hornick and Plough 2022). Within *dDocent*, Freebayes v1.3.6 (Garrison and Marth 2012) was used to generate raw variant calls and perform SNP genotyping with the modification to run without referencing population labels.

We filtered SNP loci using vcftools v0.1.16 (Danecek et al. 2011). We used an iterative filtering process as recommended by O'Leary et al. (2018). We removed loci with a minor allele count less than 3, a base quality score less than PHRED 20, a genotype call rate less than 50%, and a minimum depth less than 3 reads. Next, we visualized the distribution of missing data per individual and per locus using the—missing‐indv and—missing‐site parameters in vcftools and a custom R script. We applied an initial threshold of 81.5% missing data per individual to remove individuals that failed sequencing or sequenced poorly through the *filter_missing_ind.sh* script within the *dDocent* pipeline (*n* = 825 individuals retained). This initial cutoff is left relatively high in order to remove poorly sequenced individuals while accounting for library‐specific differences in sequencing coverage that would result in the early removal of a large subset of the fall 2020 batch, which were sequenced overall at a lower coverage than the other batches (Figure S2a). Next, to more closely target individuals with low rates of missing data at shared sites, we generated a SNP set where we retained loci called in more than 90% of individuals, with a minor allele frequency (MAF) greater than 0.01, and a missing genotype rate less than 20% within both the fall 2020 and fall 2018 libraries individually. From this SNP set, we pruned individuals with over 16% missing data based on visual inspection of a threshold for the rate of missing sites per individual for all individuals remaining in the dataset (Figure S2c; *n* = 803 individuals retained). The 803 individuals retained after applying this iterative filtering process are used in filtering SNPs for the following datasets.

Due to the unintended mixing of oyster sources prior to reef construction, we used genetic assignment to assign individual oysters back to their source population in order to address our study questions. Assignment tests, however, can be sensitive to the set of SNPs and individuals used in the analysis. Therefore, from the common set of 803 individuals and initial SNPs established from our base filtering step (that applied filters for minor allele count, base quality, etc.) we generated 12 additional SNP sets to explore the effects of MAF, missingness relative to the fall 2020 batch, and pruning for high linkage disequilibrium (LD) on downstream inferences. For all SNP sets, we retained loci called in more than 90% of individuals. We then generated three different SNP sets in which loci were retained with minor allele frequencies greater than 0.01 (SNP set 1), 0.025 (SNP set 2), and 0.05 (SNP set 3) to test the effect of MAF threshold. From each of these SNP sets, we generated an additional SNP set (SNP sets 4–6) in which we kept loci that had less than 20% missing data among fall 2020 library individuals to test whether scaling for missingness relative to the batch of individuals with the most missing sites impacted inferences downstream (e.g., Yi and Latch 2022). We then used the dDocent_filters script within the *dDocent* pipeline to filter variant calls related to allele balance at heterozygous genotypes, maximum site depth, and site/mapping quality versus site depth criteria, followed by the decomposition of variant calls into SNP and INDEL genotypes with the vcfallelicprimative command from vcflib v1.0.3. Using vcftools, we removed INDEL calls, retaining only SNP genotypes, which we then filtered to retain only bi‐allelic SNPs. Lastly, for each SNP set 1–6, we used PLINK v1.90b6.21 (Chang et al. 2015) to generate an additional SNP set (SNP sets 7–12) pruned for high LD under the following parameters: a 50 variant window, sliding five SNPs each step, and removal of one SNP from all pairs with pairwise *r* ^2^ over 0.5. Filtering outcomes for each step and SNP set are detailed in Table S1.

### 2.5 Individual Genetic Assignments and Defining Genetic Clusters

We used two complementary approaches to evaluate genetic structure in our SNP datasets and to estimate individual membership to genetic clusters (*K*) in the absence of a priori information on group membership: discriminant analysis of principal components (DAPC; Jombart et al. 2010) and sparse non‐negative matrix factorization (sNMF). Whereas DAPC is agnostic of any population genetic models and posterior membership probabilities reflect support for individual assignments to discrete genetic clusters, sNMF estimates individual admixture coefficients representing the proportion of admixture from each of the genetic clusters in the data.

DAPC was conducted using the R package adegenet (v.2.1.10; Jombart 2008) by first applying the find.clusters function to identify clusters for *K* = 1 through *K* = 10 via successive *K*‐means clustering incorporating 100 PC axes and repeated across 25 iterations. Across our 25 runs, the Bayesian Information Criterion (BIC) score was averaged and scaled by the standard deviation for each *K* value and the greatest ΔBIC value (*K*‐statistic) was estimated as in Evanno et al. (2005). This approach supported an optimal *K* = 4 (Figure S3), consistent with our prior knowledge of four distinct source hatcheries, and we used *K* = 4 to assign individuals to the best four genetic clusters in the dataset. For comparison to DAPC assignments, we performed sNMF analysis using the R package LEA (v.3.10.0; Frichot and François 2015). The sNMF results were generated for *K* = 2–10 applying an alpha parameter of 500 and 100 replicate runs per *K*. Evaluation of the “best” *K* value among these results further supported *K* = 4 (Figure S4). We extracted the highest admixture coefficient for each individual, representing the single genetic cluster for which the largest proportion of an individual's ancestry was assigned, under *K* = 4 and used these values, along with visual inspection of admixture plots, to compare to DAPC assignments. Additional details on DAPC and sNMF runs and outcomes are included in Appendix S3.

Each of the 12 SNP sets was used to generate individual genetic assignments via DAPC and sNMF, yielding a total of 24 “assignment sets.” We evaluated consistency in assignments across sets and visualized these comparisons in a single heatmap (Figure S5). Given overall high levels of concordance between sets, we chose to use the DAPC assignments generated from the LD‐pruned SNP set filtered for MAF greater than 0.01 and fall 2020 library missingness for all further analyses. We selected this dataset as a balance between prioritizing a greater number of loci, parsimony in individual assignments across SNP sets, and mitigating potential non‐biological signatures (missing data, linkage disequilibrium) that might impact clustering patterns, particularly for DAPC as a PCA‐based method.

### 2.6 Measuring Oyster Traits

To assess variation in oyster traits associated with genetic cluster identity, we recorded the following size‐related measurements for all oysters sampled in fall 2018 and 2020: shell height (mm) from the hinge to the outer shell edge; shell length (mm) from one lateral shell edge to the other at the widest point perpendicular to height; total mass (g); dry tissue and shell mass (g; tissue and shells dehydrated in drying oven for ≥ 48 h). We calculated oyster condition index as dry tissue mass × 100 divided by dry shell mass (i.e., dry tissue weight: dry shell weight ratio; Lucas and Beninger 1985; Mann 1978).

Additionally, for oysters sampled in fall 2018, we assessed infection by four common oyster parasites: the microparasites *Perkinsus marinus* and *Haplosporidium costale*, the causative pathogens of Dermo disease and SSO disease, respectively, and the macroparasites, *Cliona* spp. boring sponges and *Polydora* sp. mud blister worms. Dermo disease can impact oyster growth and reproduction (Wargo and Ford 1993; Paynter 1996), and has been associated with mass mortality events in U.S. Atlantic and Gulf coast regions (Craig et al. 1989; Ford and Smolowitz 2007). SSO disease has also been associated with mass mortality events in coastal Atlantic embayments (Andrews and Castagna 1978; Andrews 1979). Commercial breeding programs and field trials have demonstrated heritability in resistance to Dermo (e.g., Proestou et al. 2019), and that variation in resistance can arise in oysters sourced from distinct geographic regions (Brown et al. 2005; Proestou et al. 2016). While comparatively less work has been conducted with SSO, there is potential for heritable resistance to this disease (Andrews 1979) and variation in susceptibility to SSO infection has been shown among oysters from wild parental populations and a commercial hatchery source (Hanley et al. 2023). Boring sponges and mud blister worms are not often linked to mass mortalities, as compared to the microparasites, but rather are associated with decreased oyster shell integrity due to the boring and burrowing behaviors of the parasites, and with indirect negative effects on oyster growth and condition (Wargo and Ford 1993; Carroll et al. 2015). Variation in the prevalence and intensity of oyster macroparasites may also arise in response to heritable differences among source populations (Lemasson and Knights 2019; Hanley et al. 2023).

To generate estimates of microparasite prevalence, we used DNA extracted from the 32 oysters sampled per reef in 2018 and performed a polymerase chain reaction (PCR) assay protocol developed for SSO (Stokes and Burreson 2001) and a quantitative polymerase chain reaction (qPCR) assay modified for Dermo (De Faveri et al. 2009). For macroparasite prevalence, we surveyed the shells of individual oysters for physical signatures of macroparasites (i.e., holes characteristic of boring sponge; interior blisters indicating burrowing by mud blister worms). In addition to prevalence, we also report infection intensities (parasite load or concentration, per infected host) for the two most prevalent oyster parasites ( *P. marinus* and mud blister worm; reef means ± SE, 84.67% ± 4.32%, and 44.98% ± 4.50%, respectively) as an additional axis of potential variation in the response (tolerance) of oysters to parasites. Standardized intensity values for *P. marinus* were generated through the above cited qPCR protocol and were log‐transformed for all analyses and plots. For mud blister worm intensity, we quantified the overall proportion of parasite‐affected shell area using ImageJ (Abràmoff et al. 2004) following protocols from Hanley et al. (2023).

### 2.7 Statistical Analyses: Is There Non‐Random Change in Reef Genetic Composition Between 2018 and 2020?

We used generalized linear mixed models (GLMMs) to test how the relative frequencies of genetic clusters varied between sampling years on experimental reefs composed of individuals from more than one genetic cluster (*N* = 12; four reefs in fall 2018 were found to be composed of only individuals from a single genetic cluster and were excluded from all analyses). All GLMM models in this study were fit using the R package glmmTMB (Brooks et al. 2017). The response variable of reef‐level frequencies of genetic clusters (i.e., relative abundances of each distinct genetic cluster on a reef) was modeled as a function of genetic cluster identity, year, a genetic cluster identity × year interaction, experimental block, and a random effect (intercept) for reef nested within block. A significant interaction term would indicate that change in the relative frequency of genetic clusters over time varied depending on genetic cluster identity. GLMMs were fit with a betabinomial distribution to account for overdispersion and a logit link function. We applied a model selection approach, beginning with the full interaction model and dropping fixed effect terms based on the significance of likelihood ratio tests (LRT). We retained the reef random effect in all models to account for non‐independence associated with sampling multiple oyster genetic clusters from the same reef and repeated measures of the same experimental reefs over time. We evaluated whether model residuals met statistical test assumptions using the R package DHARMa (Hartig 2022). For significant interaction terms, we applied Type III sums of squares implemented through the Anova() function from the R package car (Fox and Weisberg 2019). Lastly, we performed a contrast of estimated marginal means for the effect of year on the relative prevalence of each genetic cluster using the emmeans R package (Lenth 2018). This analysis was repeated for all 24 assignment sets to address whether any differences imparted by upstream bioinformatic decisions in assigning individuals to genetic clusters impacted inferences relating to changes in the frequencies of these genetic clusters on reefs and over time (Appendix S4; Figure S6). We also ran analogous GLMMs using the raw counts of live oysters to assess consistency of genetic cluster‐specific outcomes documented in our frequency‐based approach (Appendix S4.2; Figures S7 and S8).

To provide further context to the relationships between the frequencies of each genetic cluster and the two sampling time points on individual reefs, we used Fisher's Exact Test through the fisher.test function in the R package stats (R Development Core Team 2013) to test the independence of genetic cluster identity and year of sampling. Separate contingency tables were constructed for each genetic cluster at the reef level, incorporating the counts of live oysters assigned to a focal genetic cluster (e.g., gME) and the cumulative counts of oysters assigned to the three remaining clusters (e.g., gMA, gNY, and gVA) in 2018 and in 2020. Rejection of the null hypothesis would indicate that the frequency of a genetic cluster and sampling year were related, suggesting a significant shift in the frequency of that genetic cluster over time on a given reef. This test was repeated for each genetic cluster on each reef, and *p*‐values were adjusted for multiple testing using the Benjamini‐Hochberg method (Benjamini and Hochberg 1995). We then implemented a permutation approach to evaluate whether the number of reefs showing significant shifts for each cluster was greater than expected by chance based on 1000 iterations in which sampling year labels were randomly reassigned to individuals within each reef. This analysis was repeated for all 24 assignment sets (Appendix S5; Figure S9).

Lastly, we analyzed change in reef compositional data through time with permutational multivariate analysis of variance (PERMANOVA) implemented with the function adonis2 in the R package vegan (Oksanen et al. 2018). This analysis tested the null hypothesis that reef composition, defined by the compositional frequencies of oysters from the four genetic clusters, did not differ by sampling year or experimental block (Appendix S6; Figure S10). Frequency data was first transformed (i.e., Aitchison's centered log‐ratio transformation) to address the compositional nature of the reef‐level cluster values, and statistical significance of the explanatory variables was assessed using 1000 permutation tests.

### 2.8 Statistical Analyses: How Does Genetic Diversity Differ Within and Among Oyster Genetic Clusters?

We used the primary selected SNP dataset (4679 SNPs; LD‐pruned SNP set filtered for MAF > 0.01 and fall 2020 batch missingness) to estimate genetic diversity metrics within and among genetic clusters. First, to assess the extent of genetic differentiation among the four genetic clusters, we generated pairwise *F*_ST_ estimates (Weir and Cockerham 1984) using the R package hierfstat (Goudet 2005), focusing on genetic clusters assigned from the initial samples prior to the experiment. We then estimated observed (*H*_o_) and expected (*H*_e_) heterozygosity, *F*_IS_ inbreeding coefficients, and allelic richness (*A*_r_) at the level of genetic cluster membership on each individual reef in 2018 and 2020 using the hierfstat R package. Bootstrapped confidence intervals for *F*_IS_ were generated using the boot.ppfis function applying 1000 bootstrap replicates. To assess relatedness within genetic clusters on reefs, we used the R package related v0.8 (Pew et al. 2015). Pairwise relatedness was estimated for all individuals within the same genetic cluster using the Wang estimator (Wang 2002), which has been shown to provide less biased estimates for small sample sizes, particularly among highly related individuals (Wang 2017), as would be expected within hatchery‐bred lineages. To account for dependencies in pairwise relatedness data, we applied a bootstrapping approach: for each of 1000 bootstrap iterations, individuals were resampled with replacement, and all pairwise relatedness values involving the resampled individuals were extracted. This generated a distribution of bootstrapped mean relatedness values for each genetic cluster, from which we derived a single mean pairwise relatedness estimate. For genetic clusters with only a single pairwise relatedness estimate for a reef, that single value was retained for analysis.

For each genetic diversity metric, we examined the significance of the effects of genetic cluster identity, year, a genetic cluster identity × year interaction, and block following a model selection approach using GLMMs as in the previous analyses. GLMMs were fit with a beta error distribution and logit link function, and included a random effect of reef. Metrics not bounded between 0 and 1 (i.e., *A*_r_, *F*_IS_, pairwise relatedness) were transformed to fall within these limits to fit model assumptions. Where models included significant effects of genetic cluster, block, or a significant genetic‐cluster‐by‐year interaction, we used Tukey–Kramer post hoc analyses to identify significant pairwise comparisons and correct for multiple testing.

### 2.9 Statistical Analyses: How Do Oyster Genetic Clusters Differ in Traits and in the Composition of Associated Parasite Communities?

To test whether variation in a suite of oyster traits was associated with genetic cluster identity and whether this was dependent on sampling year, we used a combination of either linear mixed‐effects models (LMM; fit with the R package lme4, Bates et al. 2015) or GLMMs in accordance with the type of oyster trait response. We applied a model selection approach as in previous analyses. For models violating assumptions, we used standard data transformations. If data transformations did not resolve violated assumptions, we applied permutation tests implemented using the permmodels function from the R package predictmeans (Luo et al. 2024) to estimate the significance of the fixed effects under 1000 permutations while preserving the random effect structure. For models including significant independent effects of genetic cluster or block, or a significant genetic cluster‐by‐year interaction, we used Tukey–Kramer post hoc analyses to examine significant pairwise comparisons.

For oyster condition index, dry tissue mass, dry shell mass, shell height, and shell length, we used LMMs to model individual traits as a function of the fixed effects of genetic cluster identity, year, a genetic cluster identity × year interaction, and block, and a random effect for reef. We removed four individual statistical outliers for condition index from 2018 and 1 from 2020 based on the Rosner Test (R package EnvStats; Millard 2013), which likely represented inaccuracies in the measurement or recording of oyster weights. The same individuals were removed for analyses of dry tissue and shell mass, as our primary interest in these traits was to provide insight into whether either trait was driving patterns in condition index. Likewise, we removed one outlier for shell height for 2020. The exclusion of outliers did not impact the significance of analyses for either trait. The residuals of the best models for condition index, shell length, and shell height all showed deviations from model assumptions (e.g., normality and homoscedasticity) that were resolved by square root transformation. Deviations associated with model residuals for dry tissue and shell mass were not resolved by standard data transformations and we applied a permutation approach to estimate model term significance as described above.

In addition to traits relating to oyster size and condition, we also modeled oyster infection by micro‐and macroparasites. Micro‐ and macroparasite presence data for individuals sampled in 2018 were converted to a binary “infection state” variable that indicated whether an oyster was infected or not infected. Individual parasite presence data was then separately analyzed for each of the four measured parasites using GLMMs with a binomial error distribution and a logit link function. We analyzed *P. marinus* intensities using an LMM, implementing a permutation approach for significance testing given the model residuals, even after data transformation, still violated multiple model assumptions. For mud blister worm intensity, we applied a GLMM approach with a beta distribution and logit link function given intensities represented the proportion of shell impacted by the parasite and were bounded between 0 and 1.

We examined whether oyster parasite community composition in 2018 (i.e., the joint prevalence of the four parasites) varied with genetic cluster identity using PERMANOVA and similarity percentage analysis (SIMPER) implemented in the R package vegan (Oksanen et al. 2018). We also examined the significance of block and reef nested within block in explaining variance in oyster parasite communities. We used a diagnostic test for multivariate dispersion via the betadisper function in the R package vegan to confirm whether these differences reflected true compositional differences (location), rather than variation in dispersion (Warton et al. 2012). To visualize significant effects on parasite communities determined by PERMANOVA, we used nonmetric multidimensional scaling (nMDS) through the metaMDS function in the R package vegan (Oksanen et al. 2018).

### 2.10 Statistical Analyses: Which, If Any, Initial Oyster Traits Are Associated With Shifts in Genetic Cluster Frequencies Over Time?

We used a multiple regression approach to examine whether the relative mortality of genetic clusters on reefs over time (approximated as change in the relative frequencies of genetic clusters on reefs between 2018 and 2020) was associated with the traits of oysters early in our experiment in fall 2018. All measured traits were averaged at the level of genetic cluster membership on each of the 12 individual reefs (*n* = 40 total combinations of the four genetic clusters on the 12 reefs). To minimize collinearity in our model, we pruned any trait pairs that were significantly correlated with a ρ rho above 0.7 to a single trait. Inclusion of *P. marinus* and mud blister worm intensities resulted in the removal of 4 out of the total 40 genetic cluster‐by‐reef observations due to zero prevalence of at least one of the two parasites. We then *z*‐standardized trait values to provide a more direct comparison of the relative importance of the traits as predictors in our multiple regression and fit the multiple regression relating the change in the relative frequencies of genetic clusters on reefs over time as a function of the standardized values of the eight oyster traits using the lm function in *R*. We assessed model assumptions using the Shapiro–Wilk test for normality and reviewing diagnostic plots. To test whether any single observations were driving observed patterns of significance in the effects of oyster traits, we calculated dfbetas values using the dfbetas function from the R package stats (R Development Core Team 2013). The dfbeta value is a diagnostic statistic that measures the influence of a specific data point on a regression coefficient by examining how much a single observation affects the estimated value of that coefficient when the observation is excluded from the model. We removed two observations that were beyond one standard error of calculated dfbeta values and re‐ran the regression. The sign and statistical significance of individual coefficient estimates did not change (Figure S11), but model fit improved and we report the outcomes of the model that excluded the two high dfbeta observations. For traits with significant effects, we generated partial regression plots (added‐variable plots) with the avPlots function in the R package car (Fox and Weisberg 2019) to visualize the bivariate relationship between the single trait and the change in reef‐level genetic cluster frequencies while accounting for the effects of all other traits in the model.

## 3 Results

### 3.1 Individual Genetic Assignments and Defining Genetic Clusters

We retained 803 (out of 885) individuals in our final SNP datasets after filtering for variant quality and library batch effects (104 initial samples, 423 from fall 2018, 241 from fall 2020, and the 35 additional samples from a small subset of reefs sampled in 2019 and 2021 excluded from downstream analyses). The number of SNPs in the 12 SNP sets ranged from 2339 to 8240 SNPs (Table S1). Our focal SNP set used in primary analyses and figures consisted of 4679 SNPs.

Assignments of individuals to the best four genetic clusters (Figure 1) produced similar results using DAPC and sNMF on each of the 12 SNP sets (Figure S5). The initial hatchery source samples were largely consistent with the estimated four best genetic clusters (Appendix S1; Figure S1), and hereafter we refer to the four estimated genetic clusters as gME, gMA, gNY, and gVA. Slight mixing of a few individuals from different genetic clusters among the hatchery source samples (Figure 1a) could be attributed to a potential mishandling/mislabeling of a few individual oysters from respective hatchery collections at the time of reef construction or samples with signatures of mixed ancestry or greater uncertainty in genetic assignment. Regardless of their relationship with specific hatcheries, the four genetic clusters represent groups of genetically differentiated individuals present on mixed reefs that we resampled through time to address our research questions.

*F*_ST_ among genetic clusters estimated from initial samples indicated moderate differentiation among all clusters (Figure S12). However, *F*_ST_ was greater between gME and gVA (0.083), gME and gMA (0.075), and gNY and gVA (0.072), whereas gME and gNY (0.051), gMA and gVA (0.053), and gMA and gNY (0.057) were more genetically similar.

### 3.2 Non‐Random Shifts in Genetic Cluster Frequencies on Reefs Over Time

**FIGURE 3:**
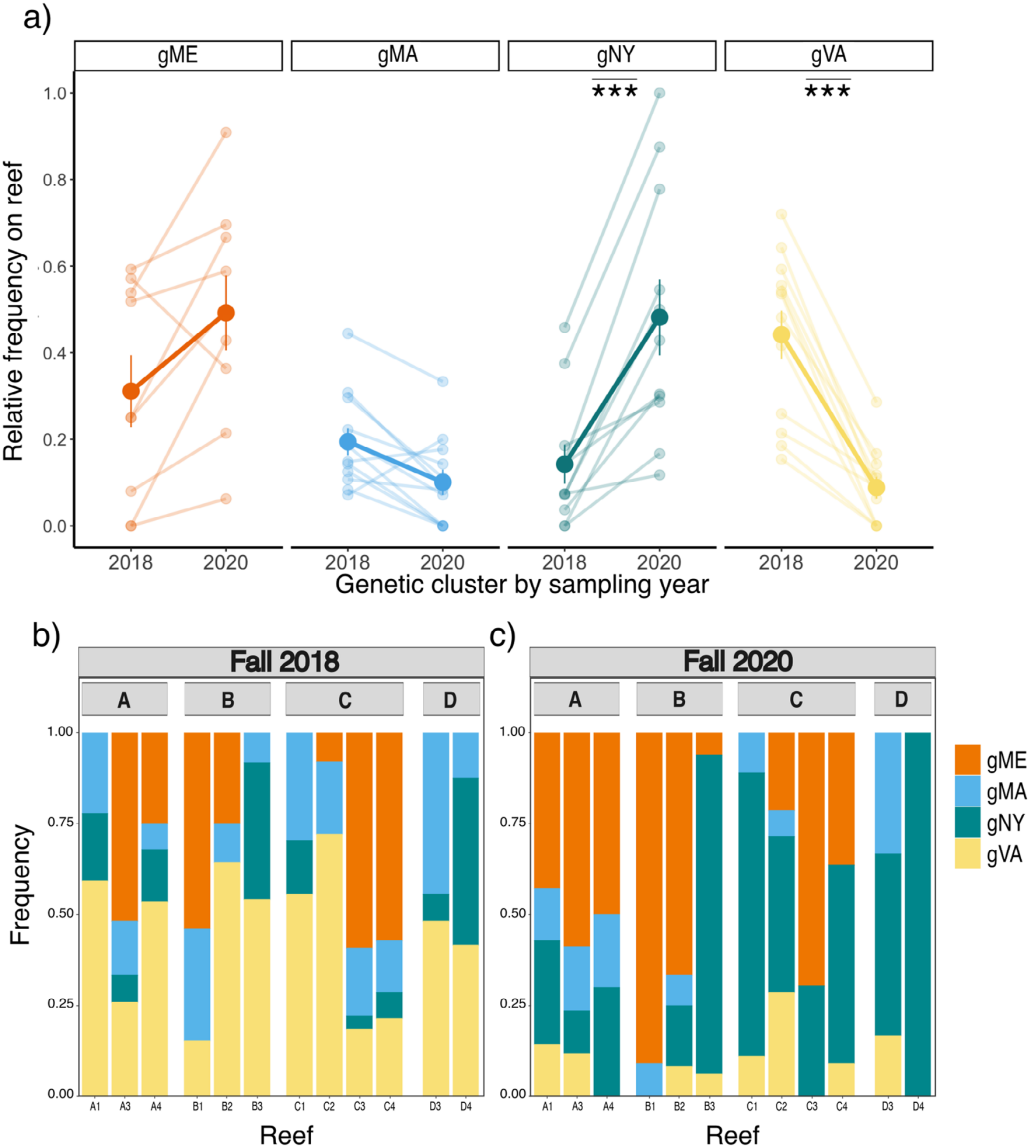
(a) Change between sampling years in the reef‐level frequencies (i.e., relative abundances) of oysters from the four genetic clusters. Bold points and lines show the average (± SE) across reefs, while fainter points and lines show individual reef‐level values. Asterisks represent significant contrasts (***: *p* < 0.001) across years for the frequencies of each genetic cluster. (b, c) Histograms representing the relative frequencies of individuals from the four genetic clusters sampled on experimental reefs in the fall of (b) 2018 and (c) 2020. Each bar depicts the composition of a single reef and the relative frequency of each genetic cluster is indicated by color. Orange = gME; blue = gMA; green = gNY; yellow = gVA.

The change in the reef‐level frequency of genetic clusters between sampling years was dependent on genetic cluster identity (Figure 3; year × genetic cluster: *X* ^2^_df=3_ = 43.776, *p* =< 0.0001; Table 1). Oysters from gVA decreased in frequency between sampling years, whereas gNY oysters increased (marginal means of year effect; gVA: estimate = −1.839 ± 0.427, *z*‐ratio = −4.311, *p* = 0.0001; gNY: estimate = 1.747 ± 0.417, *z*‐ratio = 4.194, *p* = 0.0001; Table 2). Reef‐level frequencies did not change significantly with sampling year for gME or gMA. The significance and sign of these relationships did not change with the SNP set or analytical approach used to generate individual genetic cluster assignments, except for gME, which either did not change in frequency or increased significantly between years, depending on the underlying assignment set (Appendix S4). In support of these findings, Fisher's Exact Tests indicated a significant association between genetic cluster and sampling year for most of the reefs on which gVA and gNY occurred, but not gME or gMA. These outcomes were generally consistent across the 24 assignment sets (Appendix S5; Table S2; Figure S9). Additionally, we found that the composition of reefs was strongly dependent on sampling year (PERMANOVA: year, *p* = 0.001; block, *p* = 0.116; Appendix S6; Figure S10), and that the same two genetic clusters, gNY and gVA, contributed significantly to dissimilarity between the two sampling years: gVA had higher frequency in 2018 (SIMPER, *p* = 0.004) whereas gNY was more abundant in 2020 (SIMPER, *p* = 0.016).

**TABLE 1:** Results from a Type III Sums of Squares Analysis of Deviance for the observed reef‐level frequencies (i.e., relative abundances) of live oysters from different genetic clusters and the effects of year, genetic cluster identity, and their interaction. A term for experimental reef nested in block was fit as a random intercept to control for non‐independence associated with repeated measures.

Explanatory variable	Chisq	df	*p*
Intercept	9.8684	1	0.0017**
Year	4.5550	1	0.0328*
Genetic cluster	17.2353	3	0.0006***
Year × genetic cluster	43.7758	3	< 0.0001***

*Note:* Significance codes for probability of the test statistic under the null hypothesis: ***p < 0.001, **p < 0.01, *p < 0.05, *p* < 0.1.

Abbreviations: Chisq, chi‐square test statistic; df, degrees of freedom.

**TABLE 2:** Results from estimated marginal means contrasts of the effect of year on each oyster genetic cluster. Results are averaged over the levels of the nested reef term and represented in the log odds ratio scale. *p*‐values are adjusted for multiple testing using the Bonferroni method.

Contrast	Genetic cluster	Estimate	SE	*z*‐ratio	*p*
2020–2018	gME	0.909	0.426	2.134	0.1313
2020–2018	gMA	−0.834	0.441	−1.892	0.2338
2020–2018	gNY	1.747	0.417	4.194	0.0001***
2020–2018	gVA	−1.839	0.427	−4.311	0.0001***

*Note:* Significance codes for probability of the test statistic under the null hypothesis: ***p < 0.001, **p < 0.01, *p < 0.05, *p* < 0.1.

### 3.3 Estimates of Genetic Diversity Within Genetic Clusters Over Time

**FIGURE 4:**
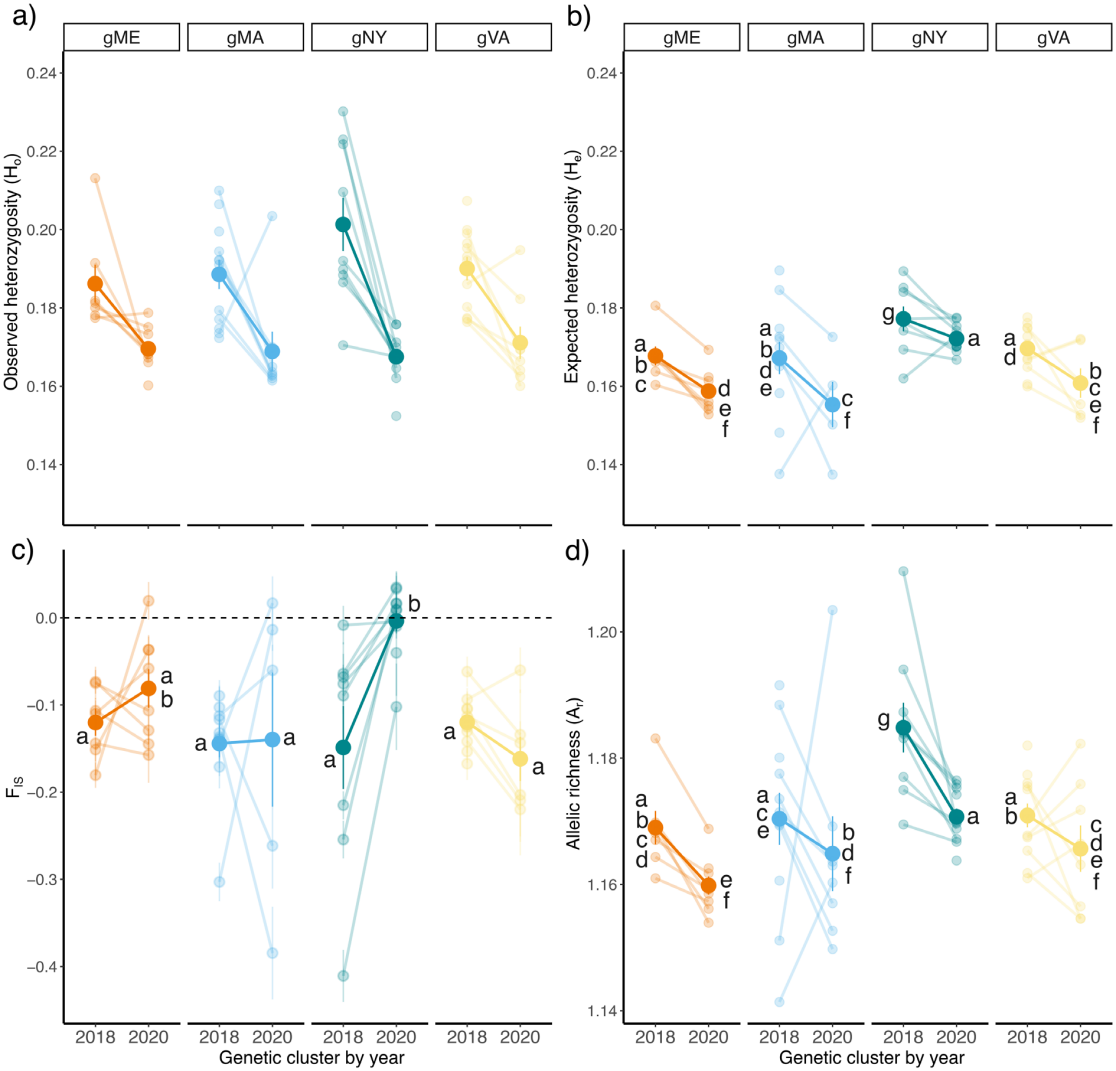
Comparisons of (a) observed heterozygosity, (b) expected heterozygosity, (c) inbreeding coefficient (F_IS_), and (d) allelic richness for genetic clusters on reefs between 2018 and 2020. Values and lines in bold represent mean values (± SE) of the genetic metrics summarized across reefs within a time point for each genetic cluster. Fainter points and lines show individual reef‐level values. Confidence intervals for reef‐level estimates of *F*_IS_ were generated from bootstrapping over loci. Letters within plots indicate significant differences (*p* < 0.05) identified with Tukey–Kramer post hoc analyses between pairwise comparisons. Orange = gME; blue = gMA; green = gNY; yellow = gVA.

Observed heterozygosity (*H*_o_) decreased between sampling years (*X* ^2^_df=1_ = 63.338, *p* < 0.0001; Table S3; Figure 4a). Similarly, expected heterozygosity (*H*_e_) and allelic richness (*A*_r_) declined through time (*H*_e_, Year: *X* ^2^_df=1_ = 15.307, *p* = 0.0001; *A*_r_, Year: *X* ^2^_df=1_ = 14.099, *p* = 0.0002; Tables S4 and S5), and also differed by genetic cluster (*H*_e_, genetic cluster: *X* ^2^_df=3_ = 25.737, *p* < 0.0001; *A*_r_, genetic cluster: *X* ^2^_df=3_ = 19.520, *p* = 0.0002; Tables S4 and S5). Both *H*_e_ and *A*_r_ were higher for gNY than for all other groups (Figure 4b,d; Tukey–Kramer contrast, *p* < 0.05). We found a significant interaction between year and genetic cluster for *F*_IS_ (year × genetic cluster: *X* ^2^_df=3_ = 15.724, *p* = 0.0013; Table S6), reflecting the positive shift over time in *F*_IS_ for gNY as compared to the other genetic clusters (Figure 4c). The average pairwise relatedness among individuals within genetic clusters varied with cluster identity and sampling year (year × genetic cluster: *X* ^2^_df=3_ = 10.1807, *p* = 0.0171; Table S7; Figure S13). In fall 2018, mean relatedness within genetic clusters did not differ by genetic cluster identity (Tukey–Kramer contrast, *p* < 0.05). In fall 2020, average relatedness had decreased significantly for gNY (Tukey–Kramer contrast, *p* < 0.05), but did not change consistently among individuals within the other genetic clusters. Broader consideration of results relating to genetic diversity is included in Appendix S7.

### 3.4 Variation in Oyster Traits and in the Composition of Associated Parasite Communities by Genetic Cluster

**FIGURE 5:**
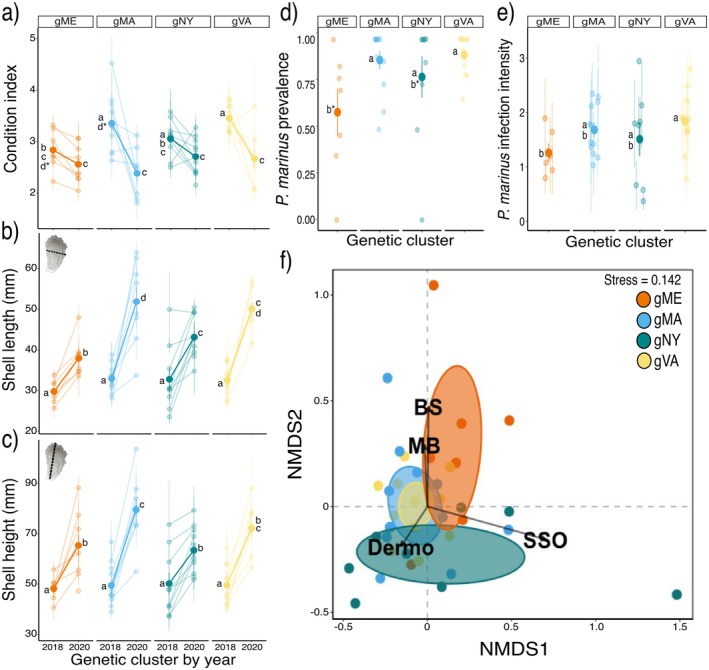
Plots depicting variation by genetic cluster identity in oyster (a) condition index, (b) shell length, and (c) shell height in fall 2018 and fall 2020, and in (d) *P. marinus* infection prevalence and (e) log‐transformed intensity, and (f) the composition of oyster parasite communities in fall 2018. For panels a‐e, bold points and lines show the average trait values for genetic clusters (± SE) summarized across reefs by sampling year, while fainter points and lines show individual reef‐level averages (± SE). Letters within these plots indicate significant pairwise comparison (*p* < 0.05) identified with Tukey–Kramer post hoc analyses. Letters with an asterisk (*) indicate pairwise comparisons that are marginally significant (0.05 ≤ *p* ≤ 0.1). Panel (f) shows a nonmetric multidimensional scaling (nMDS) plot depicting prevalence of microparasites *P. marinus* (Dermo), *H. costale* (SSO), and macroparasites *Polydora* sp. (mud blister worm, MB) and *Cliona* spp. (boring sponge, BS) for oysters from different genetic clusters in fall 2018. Ellipses indicate 95% confidence intervals for genetic cluster membership. Data points represent the relative composition of parasite communities sampled from oysters of different genetic clusters on each of the 12 restored reefs (*n* = 40 genetic cluster*reef combinations). Bolded segments with arrows represent correlations between the nMDS ordination axes and the prevalence of the four representative oyster parasites. Orange = gME; blue = gMA; green = gNY; yellow = gVA.

Oysters from all genetic clusters significantly increased in average shell height and length between sampling years (Tukey–Kramer contrast, *p* < 0.05; Figure 5b,c), yet the slope of this relationship depended on genetic cluster identity (Shell height, year × genetic cluster: *F*_df=3_ = 2.7298, *p* = 0.0434; Shell length, year × genetic cluster: *F*_df=3_ = 4.8803, *p* = 0.0024; Tables S8 and S9). There were no differences associated with genetic cluster identity for shell height or length in 2018, but oysters from gME were of significantly smaller shell length than oysters from all other genetic clusters by fall 2020 (Tukey–Kramer contrast, *p* < 0.05; Figure 5b,c). Oysters from gMA were of significantly larger shell height and length than oysters from gME and gNY by fall 2020 (Tukey–Kramer contrast, *p* < 0.05). We found a marginally significant interaction between year and genetic cluster for condition index (year × genetic cluster: *F*_df=3_ = 2.5351, *p* = 0.056; Table S10), likely representing differences in the condition of oysters among genetic clusters in fall 2018 (with gME lower than gVA) that were no longer present in 2020 (Figure 5a). Patterns in condition index were not clearly related to either dry tissue or shell mass alone (Appendix S8).

Parasite community structure varied significantly by genetic cluster (PERMANOVA: genetic cluster, *p* = 0.020; Figure 5f), but not by block nor reef nested within block. A test for multivariate dispersion confirmed these differences were due to true compositional distinctions rather than differences in within‐group variability (*p* = 0.247). The prevalence of infections by macroparasites (mud blister worm or boring sponge) was correlated with belonging to gME, whereas fewer infections by these parasites and a greater prevalence of *P. marinus* infections was associated with cluster gNY. In addition, gMA generally had a higher prevalence of mud blister worm infections than gNY (SIMPER, *p* = 0.010), and lower boring sponge prevalence than gME (SIMPER, *p* = 0.015).

In univariate analyses of individual parasite infection, the presence of *P. marinus* infections varied significantly with genetic cluster identity (Genetic cluster: *X* ^*2*^_df=3_ = 26.692, *p* < 0.001; Block: *X* ^2^_df=3_ = 19.613, *p* = 0.0002, Figure 5d; Table S11). Oysters from gME had significantly fewer *P. marinus* infections than gMA and gVA (Tukey–Kramer contrast, *p* < 0.05). Infections by boring sponge varied marginally among genetic clusters (Genetic cluster: *X* ^2^_df=3_ = 6.6539, *p* = 0.0838; Figure S14c; Table S12): gME oysters tended to have more boring sponge infections compared to gNY (Tukey–Kramer contrast, *p* = 0.065). The presence of mud blister worm infections was more closely predicted by experimental block alone (Block: *X* ^2^_df=3_ = 15.684, *p* = 0.0013; Table S13). Neither genetic cluster nor block explained a significant amount of variance in the presence of infections by *H. costale*.

Intensity of *P. marinus* infections was explained by the main effects of genetic cluster and experimental block (Monte Carlo permutation tests; genetic cluster: *F*_df=3_ = 3.893, *p* = 0.007; Block: *F*_df=3_ = 3.177, *p* = 0.031; Figure 5e; Table S14): gME oysters had significantly less intense infections than those from gVA (Tukey–Kramer contrast, *p* < 0.05). Neither genetic cluster identity nor block described significant variance in mud blister worm intensity.

### 3.5 Relating Initial Oyster Traits to Shifts in Genetic Cluster Frequency

**FIGURE 6:**
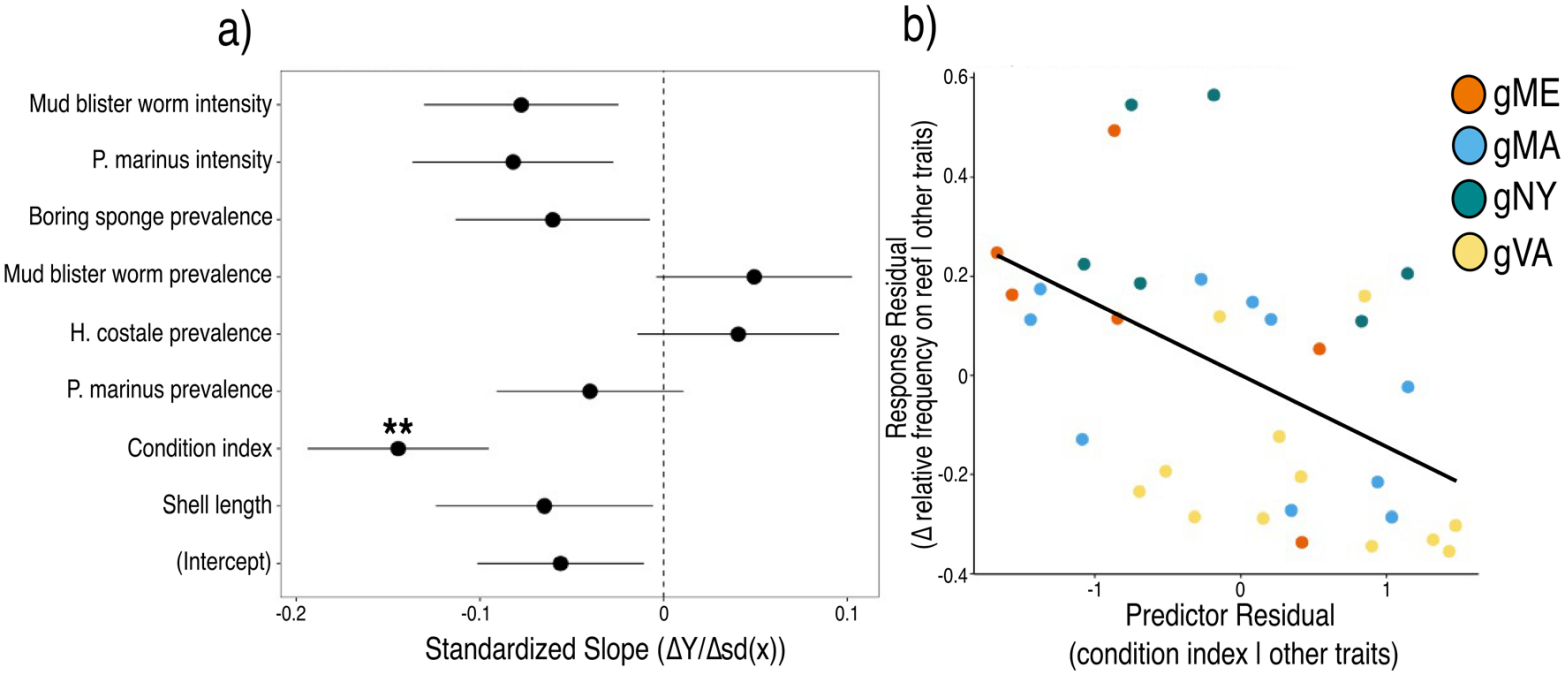
(a) Standardized coefficients from a multiple regression of the relationship between the change in the reef‐level frequency of genetic clusters and the average values of eight oyster traits from these groups in fall 2018. The model was fit to normalized (z‐transformed) trait values to generate standardized estimates of the coefficients for all traits. Significance for the probability of the t‐statistic of each coefficient is indicated as: ***p < 0.001, **p < 0.01, *p < 0.05, *p* < 0.1. (b) Partial regression plot of the relationship between the change in the relative frequency of genetic clusters on individual reefs over time and the mean condition index of groups on reefs in 2018. The partial regression accounts for the effects of the seven additional oyster traits used as explanatory variables in the multiple regression. Orange = gME; blue = gMA; green = gNY; yellow = gVA.

Oysters from genetic clusters with higher condition index in fall 2018 tended to experience greater decreases in reef‐level frequencies between sampling years (estimate ± SE = −0.145 ± 0.049; *X* ^2^_df=1_ = 8.567, *p* = 0.003; Figure 6a,b). There were no statistically significant relationships between the other seven measured traits and the change in the reef‐level frequencies of oysters (Figure 6a).

## 4 Discussion

Our experimental oyster restoration highlights the value of embedding evolution‐minded experiments into ecological restoration efforts (Stockwell et al. 2016; Bailey et al. 2021; Hendry et al. 2024; Nadeau et al. 2024), as a means to gain fundamental insights into eco‐evolutionary processes and to improve restoration success. We documented strong shifts in the relative abundance of oysters from certain genetic clusters on our restored reefs over the span of just 2 years. The absence of natural recruitment in this system during our study (Barrett et al. 2024) and the consistency in the direction of changes across individual reefs suggest that these shifts in reef demographics can be attributed to differential mortality, rather than gene flow or genetic drift. The association between higher condition at the start of the experiment and higher relative mortality over time suggests an unexpected relationship between this trait, or traits associated with condition early in the experiment, and oyster survival, potentially mediated through increased predation rates. Our findings that the genetic identities of individuals can play a significant role in patterns of mortality on restored reefs on restoration‐relevant time scales complement small‐scale experimental studies demonstrating ecological effects of genetic variation in oysters (Hanley et al. 2016; Hughes et al. 2019). This work also illustrates how genetic sampling can be integrated into experimental restoration design to explore eco‐evolutionary dynamics over the course of a restoration project.

While the persistent shifts that we observed in the reef‐level frequencies of genetic clusters provide an indication that selection has occurred, selection ultimately acts upon variation in fitness‐related traits among these groups. Of the traits we measured early in our experiment, condition index was the only trait significantly associated with patterns of relative mortality: genetic clusters with higher condition in 2018 (e.g., gVA) showed greater reductions in reef‐level frequencies over time. However, we observed meaningful variation in other traits, including parasite infection and shell size, associated with genetic cluster identity that seemed to partially align with patterns in survival. For instance, oysters from gME, which did not shift significantly in reef‐level frequency over time, tended to have fewer and less intense infections by the most prevalent parasite, *P. marinus* , relative to oysters from gVA, which declined the most in reef‐level frequency. Yet, oysters from gNY, which increased consistently in reef‐level frequency over time, had similarly high prevalence and intensity of *P. marinus* infection to gVA, as well as gMA. Additionally, although shell height and length were similar among genetic clusters in 2018, oysters from gVA and gMA tended to have larger shells compared to oysters from gME and gNY by 2020. It is important to note that oyster growth and mortality happened concurrently in this experiment and thus are confounded in this respect; this issue, referred to as the invisible (or hidden) fraction, results from the portion of a population that is absent when a trait of interest is measured as a result of viability selection acting on the population before the measurement occurs (Grafen 1988; Hadfield 2008). For instance, oysters from the different genetic clusters may have grown at the same rate, but due to higher selective mortality of smaller gVA oysters, the mean size of gVA as measured in 2020 would be higher than the other groups even with equal growth rates. Ultimately, genetic cluster identity, early oyster condition index and disease patterns, oyster growth, and differential mortality were all intertwined in our experiment and, to some collective extent, modulated the shifts in reef demographics observed in our experiment.

Because the oysters used in this experiment were not reared through multiple generations in a common environment, it is difficult to differentiate among potential drivers of trait variation. Variation in condition and parasite susceptibility among genetic clusters early in the restoration likely represented either heritable or environmental hatchery effects present upon sourcing, given that oyster larvae from the various hatcheries were received and processed by the same Rhode Island facility, maintained on nearby oyster grower leases on similar time scales, and outplanted together on mixed restoration reefs. Commercial hatcheries often apply selective breeding programs that favor traits that enhance aquaculture production, such as rapid growth and disease resistance. Variation in the degree of selection among the commercial hatchery stocks for said traits could have yielded the observed variation in oyster traits. Alternatively, maternal or early environmental hatchery effects stemming from hatchery‐specific rearing or shipping conditions could underlie the trait patterns we detected. While we are limited in pinpointing the exact mechanism for variation in traits among the genetic clusters, our sourcing approach reflects current restoration practice involving hatchery production and supplementation (Barrett et al. 2024), and thus the implications of source‐related variation in performance remain relevant to outcomes in similar restoration efforts and management contexts.

Beyond the potential origins of oyster trait variation, we propose a few possible interpretations for the observed relationship between early condition index and selective mortality. Condition index, defined as the ratio of tissue mass to shell mass (Mann 1978), is increased in the presence of greater tissue mass and/or thinner shells. Negative selection on condition index may have been driven by heightened predation on juvenile oysters with reduced shell thickness and/or higher tissue mass (e.g., Johnson and Smee 2012; Robinson et al. 2014). If constitutive differences in condition existed among genetic clusters upon sourcing, variation in survival could reflect cluster‐specific susceptibility to predation. Alternatively, oysters and other bivalves can respond to predation cues by producing thicker or harder shells (Robinson et al. 2014; Scherer et al. 2018; Belgrad et al. 2021) and/or reducing feeding activity (Smee and Weissburg 2006; Naddafi et al. 2007; Johnson and Smee 2012), both of which would reduce condition index. Thus, differences in survival may also have reflected variation in the capacity to induce these plastic shifts in morphology or behavior in response to predation risk (Bible et al. 2017; Kimbro et al. 2022). Future oyster restoration experiments that manipulate and measure predation pressure could help clarify the role of predation as a selective force in oyster restoration contexts.

A second possibility is that patterns in mortality reflect tradeoffs associated with traits selected for aquaculture (“cultivation”). Condition index is associated with a suite of traits considered “desirable” to oyster growers and consumers (e.g., increased growth rate, mean individual weight) that are often under selection in commercial hatchery programs (de Melo et al. 2016; Allen Jr et al. 2021)—yet, these traits may not confer survival advantages outside of hatchery/aquaculture conditions. Hatchery breeding programs can inadvertently reduce performance in more natural settings through genetic correlations with maladaptive traits or adaptation to hatchery conditions (i.e., domestication selection)—a phenomenon well‐documented in salmonids (Araki et al. 2008), and to a limited degree in oysters (e.g., McFarland et al. 2020). While few studies have examined these tradeoffs directly, lineages selected for rapid growth—a common cultivation‐associated phenotype—have been shown to have lower metabolic rates and greater energy allocation to growth (Sydney rock oyster, *Saccostrea glomerata*, Bayne 2000; Bayne 2004; eastern oyster, Pernet et al. 2008), but potentially reduced survival under thermal stress (Sydney rock oyster, McAfee et al. 2017). In other systems, fast growth has been linked to tradeoffs with stress resistance and immune function (Mangel and Stamps 2001). In our experiment, higher mortality among the larger, high‐condition gVA oysters may indicate a tradeoff with another fitness‐associated trait, such as reduced tolerance to parasitic infection. Conversely, the persistence of gNY oysters, which were of smaller size but had similarly high *P. marinus* infections, may suggest a tolerance‐growth tradeoff where more tolerant genotypes invest less in growth. However, because growth and mortality occurred simultaneously in our experiment, trait measurements from later timepoints are subject to the “invisible fraction” problem described above (Grafen 1988; Hadfield 2008), limiting our ability to disentangle patterns in trait variation from differential mortality. More temporally resolved sampling and incorporation of physiological or metabolic traits in future studies could help disentangle the role of potential growth‐related tradeoffs.

Regardless of the driver underlying selection on condition index, the pattern is somewhat counterintuitive: typically, condition index is considered a bioindicator of oyster health (Lucas and Beninger 1985), yet higher condition index at the start of the experiment was correlated with higher relative mortality over time. Other studies of selection in restoration have similarly called attention to a potential mismatch between traits perceived to be of benefit and prioritized in restoration (e.g., large initial size) versus realized outcomes. For example, Bailey and Kinnison (2010) estimated selection gradients on juvenile Atlantic salmon (fry) size following hatchery propagation and stocking with river‐specific broodstock. They found strong directional selection on fry size in five of the six isolated river populations, suggesting that the current distribution of fry size‐at‐stocking seemed poorly matched to existent habitat conditions. Similarly, in a resurrection ecology‐style approach comparing the distributions of traits in restored populations against that of the original seed sources used to sow the restorations, Kulpa and Leger (2013) showed consistently higher fitness for smaller plants and seed size among experimentally sown restoration grasses. This combination, the authors noted, was in contrast to traits typically associated with increased production in agricultural settings (i.e., large plant and seed size) and typically prioritized for prairie restoration (Kulpa and Leger 2013). These examples highlight the value of directly evaluating selection in restoration and management efforts for challenging accepted, yet largely untested, wisdom informing standard restoration practice.

Current practice for restoration sourcing often assumes “local is better”, under the premise that local sources are better matched to selective pressures in the local environment (McKay et al. 2005; Leimu and Fischer 2008; Breed et al. 2018). In our study, the genetic cluster associated with the most geographically distant hatchery showed the greatest decline in reef frequency, indicating potential maladaptation to the restoration site. Conversely, the genetic cluster associated with the most local hatchery increased in frequency, suggesting these oysters were better adapted than those from the most distant hatchery. Similar trends have been documented in previous transplants of selectively bred oyster lines sourced from hatcheries along the U.S. Atlantic coast to estuaries in the Northeast United States, where more southern‐sourced lines sometimes performed more poorly compared to local lines (Rawson and Feindel 2012; Proestou et al. 2016), though not universally (Proestou et al. 2016). Similarly, hatchery geographic distance did not fully explain the patterns of differential mortality in our study: gME, the other more “distant” hatchery maintained high relative abundances on reefs over time.

Several factors may explain the inconsistency of geographic distance as a predictor of source performance in our study. First, hatchery location may not be a perfect proxy for the origin of sourced oysters—broodstock collections may come from other regions or reflect mixed ancestry due historical translocations (Miller 2000; Hoos et al. 2010). Indeed, pairwise *F*_ST_ values among genetic clusters do not uniformly support the assumption that hatchery location equates to source population origin (Figure S12): gMA (Massachusetts) was most genetically similar to gVA (Virginia), while gME (Maine) was more similar to gNY (New York) than to gMA. Moreover, environmental similarity and selective pressures do not always scale with geographic distance or latitude (e.g., Helmuth et al. 2002). Further, there can be substantial small‐scale variation in these same selective pressures (e.g., by reef depth; Fodrie et al. 2014; Malek and Byers 2017). Thus, genetic and/or environmental similarity between potential sources and a restoration location or recipient population may be more reliable predictors of source performance than geographic distance alone. Lastly, oysters in commercial hatchery programs are notably a step removed from natural selective pressures, and variation in performance among sources could instead primarily reflect variation in hatchery practices unexplored in this study. We echo calls for greater collaboration among commercial facilities (e.g., hatcheries, nurseries), restoration practitioners, and research scientists regarding the production and sourcing of restoration material (e.g., Hughes et al. 2019; Barak et al. 2022) to facilitate experimental and adaptive ecological restoration (Broadhurst et al. 2017; Goodale et al. 2023; Nadeau et al. 2024). Such partnerships will be particularly valuable as interest grows in incorporating “pre‐adapted” variation into restoration programs (e.g., assisted gene flow; Aitken and Bemmels 2016; Nadeau et al. 2024; Twardek et al. 2023) and, separately, management frameworks that use commercial production as a tool for species restoration (e.g., “conservation aquaculture”; Froehlich et al. 2017; Wasson et al. 2020; Ridlon et al. 2023).

There is an increasing need to understand the factors that contribute to restoration success in order to meet the wave of global interest in restoration programs and to maintain restored populations into the future. Ultimately, most of our experimental reefs were driven to low oyster abundances by the end of our study, demonstrating that repeated seeding may be needed to sustain reefs in this system. Amidst overall reductions in abundance, however, we observed consistent shifts in reef composition driven by differential mortality among hatchery sources, with one source clearly performing worse. However, the generalizability of these patterns across other restoration sites and with other hatchery lines remains uncertain. Given inherent uncertainty regarding the “best” source material to use for any particular restoration project, we recommend that restoration programs, both operational and experimental, use a mix of diverse sources (e.g., regional or composite provenancing; Broadhurst et al. 2008; Bucharova et al. 2019) as a form of bet‐hedging (Lesica and Allendorf 1999) assuming that sources may be variably appropriate under different restoration contexts. Coupling this approach with repeated seeding offers multiple opportunities for managers to incorporate feedback on source performance and adaptively refine restoration strategies.

Several aspects of the experimental design warrant consideration when interpreting our results. Unequal initial representation of genetic clusters on reefs and realized distributions that were not truly random limited our ability to assess survival rates independently of starting frequency. Although we addressed this by analyzing proportional shifts, confirming directional patterns across reefs, and applying multiple analytical approaches, we cannot fully exclude the influence of initial abundance differences. Likewise, we cannot completely rule out neutral demographic processes (e.g., ecological drift) as a partial contributor to observed patterns. Nonetheless, the consistent trends across reefs and analytical methods, alongside the association with condition index, provide compelling support for a selective mechanism. Future experiments using additional hatchery lines and/or conducted across environmentally distinct sites would help evaluate the generality of our findings and clarify how external conditions shape lineage‐specific performance.

While the value of integrating consideration of evolution into restoration programs is becoming more widely appreciated (Wainwright et al. 2018; Jordan et al. 2024), most programs are conducted in the absence of direct insight into the evolutionary processes that might promote or compromise restoration goals. Evolutionary biologists, through the application of tools and approaches to assess evolutionary patterns and processes, have the capacity to help in providing such insight. At the same time, restoration practitioners have detailed knowledge of the system at hand that can inform our fundamental understanding of evolution in nature. Considering active efforts to increase the scale of ecological restoration globally (e.g., UN Global Decade on Ecosystem Restoration), we join calls for scaled, controlled eco‐evolutionary experiments embedded within restoration plantings (Stockwell et al. 2016; Broadhurst et al. 2017; Bailey et al. 2021; Nadeau et al. 2024) and greater support for the establishment of collaborations between restoration managers and evolutionary research scientists.

## Conflicts of Interest

The authors declare no conflicts of interest.

## Supporting information


Appendices S1‐S8 and Figures S1–S14.

Tables S1–S18.

## Data Availability

Data for this study is available through the Northeastern University Digital Repository Service at: https://hdl.handle.net/2047/D20775066. Raw sequence reads are deposited in the NCBI SRA (BioProject ID PRJNA1280068) at: https://www.ncbi.nlm.nih.gov/sra/PRJNA1280068.
