# Supplementary material for: Non‐Random Mortality in an Experimental Oyster Restoration
Source: Evol Appl. 2025 Jul 6;18(7):e70128. doi: 10.1111/eva.70128 (PMC12229816; doi:10.1111/eva.70128)

Supplementary Materials: Truskey et al. Non-random mortality in an experimental oyster restoration. *Evolutionary Applications*

Appendix Sections

Section S1. Unintended mixing of hatchery-sourced samples prior to reef construction
Section S2. Preparation protocol for ddRADseq library
Section S3. Additional information on DAPC and sNMF run parameters and outcomes
Section S4. Concordance in analyses examining shifts in reef-level genetic cluster frequencies over time
Section S4.1. Overall concordance in GLMM output evaluating shifts in genetic cluster frequencies across 24 assignment sets
Section S4.2. Comparison of approaches using absolute, versus relative, reef-level abundances shows concordant but weaker signal
Section S5. Concordance in the outcomes of Fisher's Exact Tests on the relationship between genetic cluster frequencies and sampling year across the 24 assignment sets
Section S6. Additional support for shifts in the genetic composition of reefs over time from distance-based methods
Section S7. Discussion of results relating to within-cluster genetic diversity
Section S8. Variation between genetic clusters in fall 2018 condition index not independently explained by either dry tissue or shell mass

Supplementary Figures

Figure S1. PCAs depicting unintended mixing of hatchery-sourced samples prior to reef construction
Figure S2. Plots showing batch library-specific bias in distribution of missing data and associated filtering treatment approach
Figure S3. Plots depicting process of identifying optimal K for DAPC
Figure S4. Plots depicting process of identifying optimal K for sNMF
Figure S5. Concordance of individual assignments to genetic clusters between DAPC and sNMF for the 24 genetic assignment sets
Figure S6. Concordance across 24 assignment sets in average reef-level changes in genetic cluster frequencies and estimated year effects.
Figure S7. Reef-level changes in absolute counts of oysters by genetic cluster and distribution of genetic cluster counts across reefs in 2018 and 2020.
Figure S8. Concordance across 24 assignment sets in average reef-level changes in genetic cluster counts and estimated year effects.
Figure S9. Concordance in outcomes of Fisher's Exact Test and associated permutations evaluating changes in genetic cluster frequencies over time for the 24 genetic assignment sets
Figure S10. Non-metric multidimensional scaling (nMDS) plot depicting shifts in oyster reef genetic composition over time
Figure S11. Effect of pruning dfbeta outliers on the regression of genetic cluster frequency changes on oyster traits
Figure S12. Pairwise genetic differentiation (F_{ST}) among genetic clusters
Figure S13. Change in relatedness within genetic clusters over time
Figure S14. Single oyster parasite infection patterns by genetic cluster identity

Appendix Sections

Section S1. Unintended mixing of hatchery-sourced samples prior to reef construction

Analysis of initial juvenile oyster spat-on-shell samples received from local growers corresponding to the various oyster hatchery sources revealed an early, unintended mixing of some of the hatchery sources prior to reef construction (Figure S1). Of the 6 hatchery-source*oyster-grower combinations (Grower 1-MA; Grower 2-ME; Grower 2-NY; Grower 2-VA; Grower 3-NY; Grower 3-VA), PCAs show consistent patterns in mixing among the hatchery labels, primarily associated with samples from Grower 2 (Figure S1; dashed ellipses in PCAs in panels a and b). Notably, these patterns were consistent between iterations of these analyses employing only the initial hatchery samples (n=5605 filtered SNPs, 110 individuals) and genotype likelihood approaches (via ANGSD), and the final filtered dataset in which we combined the 3 ddRAD libraries (n= 4476 filtered SNPs; 803 individuals from the 3 libraries with only the 104 initial library individuals visualized) and used genotype calls (via dDocent/FreeBayes) (Figure S1). This concordance provides further support that this apparent pattern does not reflect technical artifacts in our combined 3-batch ddRAD library, but rather mislabeling/unintended mixing of respective hatchery lineages at some point prior to reef construction.

The sample labeled by Grower 2 as ME (Grower 2-ME), the hatchery from ME that was exclusive to Grower 2, appears to be mislabeled and more likely represented a mixture of hatchery sources. We inferred that these oysters are primarily NY, in that they are clustering tightly with the NY-labeled group from Grower 3. The unique Grower 2 cluster labeled as VA (Grower 2-VA) also appeared mislabeled, since it did not cluster with the other VA source (Grower 3-VA) and rather, more likely represented ME as the only putative unique Grower 2

genetic cluster. Excluding the hatchery source labeled samples supplied from Grower 2 that were unintended multi-source mixtures (i.e., Grower 2-ME and Grower 2-NY, as labeled), the initial hatchery source samples were largely consistent with the estimated four best genetic clusters (Figure S1c) and we refer to the 4 estimated genetic clusters throughout the text as gME, gMA, gNY, and gVA.

Section S2. Preparation protocol for ddRADseq library

We prepared each of the three batches of genomic ddRAD libraries following protocols in Parchman et al. (2012). Briefly, the main steps of the protocol included, (1) restriction digest with two restriction enzymes, EcoRI and MseI; (2) ligation of subsequent DNA fragments with unique 8- to 10-base pair indexed adaptors (barcodes); (3) the amplification of restriction-ligation products in two independent PCRs using standard Illumina primers; and (4) the pooling of all amplicons. Pooled library products were then sent to the respective sequencing facilities detailed below.

The three batches were prepared and sequenced separately: the first batch (initial hatchery samples) was prepared in 2020 and sent for to the Tufts University Core Facility Genomics (TUCF-Genomics; Boston, MA) for sequencing, the second batch (2018 samples) was prepared in 2021 and sent to the University of Texas at Austin Genomic Sequencing and Analysis Facility (UT-GSAF; Austin, TX) for sequencing, and the final third batch (2020 samples, plus extra samples from 2019) was prepared concurrently to the second batch in 2021, but sent to TUCF-Genomics for sequencing. At the two sequencing facilities, the ddRAD library batches underwent an additional size-selection step for 300- to 500-base pair fragments using the Blue Pippin Prep (Sage Science, Beverly, MA, USA). All libraries were sequenced with 100-bp

single-end sequencing on an Illumina platform. The initial and fall 2020 batches were each separately sequenced on a single lane on the Illumina HiSeq 2500 platform at TUCF-Genomics and the larger fall 2018 batch was sequenced on two lanes on the Illumina NovaSeq 6000 platform at UT-GSAF.

Section S3. Additional information on DAPC and sNMF run parameters and outcomes

In DAPC analyses run on our data, there was no obvious lowest value (“elbow”) representing a “best K” in the resultant BIC plots within a reasonable range of K (Figure S3a). The expectation that the optimal K is represented as the outcome with the lowest BIC value, however, may not hold for complex genetic datasets, for instance involving stepping stones or isolation-by-distance (adegenet, find.clusters R documentation), and in such cases BIC often continues to decrease beyond the “best K”. In our case, this finding was not necessarily surprising: we have a relatively large number of individuals in our dataset, the oyster genome is in general associated with a relatively high degree of polymorphisms, our SNP data were produced in separate sequencing batches (although we attended to obvious batch effects), and hierarchical structure beyond regional hatchery sources, for instance corresponding to different oyster family groups within a hatchery, may be present in our dataset. An alternative approach to selecting an optimal K value is to consider the degree of change in BIC between successive values of K and retain the K value differentiating steep decreases in BIC from more moderate declines. This approach assumes that the greatest amount of genetic differentiation in our data corresponds with broad-scale geographic variation among the regional source hatcheries, whereas subsequent axes of variation beyond these (e.g., families, cryptic batch effects) will represent finer-scale changes in genetic structure and/or variation among individuals.

Across our 25 DAPC runs, BIC scores were averaged and scaled by the standard deviation for each K value, and these values were used to calculate the largest ΔBIC value (K-statistic) (Figure S3c-d), as in Evanno, Regnaut, and Goudet (2005). This approach supported retaining K=4, consistent with our prior knowledge of 4 distinct source hatcheries, and we used this value to assign individuals to the best 4 genetic clusters in the dataset. The optimal number of PCs provided for the final DAPC run was determined as the number of PCs associated with the lowest root mean squared error in individual assignments returned from the cross-validation function xvalDapc, which assessed assignments incorporating 1 to 100 PCs on a training set of 90% of individuals and 100 replicates carried out at each level of the retained PCs.

As with DAPC, the "best K" for sNMF analyses was not immediately obvious from sNMF generated cross-entropy (CE) values (Figure S4a). Applying a similar approach to assess the largest ΔCE values, we found additional support for K=4 (Figure S4c). Thus, we report sNMF results using K=4. We extracted the highest individual admixture coefficient output from sNMF for each individual, representing the cluster with the highest proportion of an individual's ancestry, to compare against DAPC-assigned cluster membership assignments.

Section S4. Concordance in analyses examining shifts in reef-level genetic cluster frequencies over time

S4.1. Overall concordance in GLMM output evaluating shifts in genetic cluster frequencies across 24 assignment sets

For all 24 assignment sets, the effect of sampling year on the frequencies of genetic clusters on reefs was significant and consistently negative for gVA and positive for gNY (Figure S6). Reef level-frequencies did not change significantly with sampling year for genetic cluster gMA across

any assignment sets. The significance of shifts in the reef-level frequencies of gME, however, depended on the underlying assignment set: we found a significant positive shift in the frequency of gME in all but one of the analyses using DAPC assignments (11 out of the 12 assignment sets) as compared to only a single sNMF-generated sets (only 1 out of 12 assignments sets).

S4.2. Comparison of approaches using absolute, versus relative, reef-level abundances shows concordant but weaker signal

To complement our main analyses based on relative abundances (i.e., frequencies), we also fit generalized linear mixed models (GLMMs) to the absolute counts of live oysters from each genetic cluster. These models used a negative binomial distribution to account for overdispersion in count data. Results mirrored those from the frequency-based approach: changes in live oyster counts over time depended on genetic cluster identity (year*genetic cluster: $X^2_{df=3}$= 46.331, P<0.001; Table S15; Figure S7-8). The counts of oysters from gVA decreased significantly between sampling years, whereas counts of gNY oysters marginally increased (marginal means of year effect; gVA: estimate= -2.0513 ± 0.336, z-ratio = -6.111, p<0.0001; gNY: estimate= 0.7885 ± 0.301, z-ratio = 2.615, p=0.036). The counts of oysters from gMA also declined significantly (gMA: estimate= -1.2368 ± 0.349, z-ratio = -3.546, p=0.002; Supplementary Table S16), despite gMA not showing significant shifts in our primary frequency-based analyses.

However, the significance of the increase in gNY counts was sensitive to the assignment method. In GLMMs based on sNMF assignments, 10 of 12 assignment sets showed a significant or marginal increase in gNY counts, while only 1 of 12 DAPC-based sets did. In contrast, the decline in gVA and gMA counts was consistent across all assignment sets. This contrasts with the

consistency of outcomes using frequencies rather than absolute counts reported in Supplementary Appendix S4.1, where we found consistent shifts in the frequency of gVA (decrease) and gNY (increase) across assignment sets and no significant shift in the frequency of gMA. These inconsistencies likely reflect a key limitation of using absolute counts in our study: total reef-level oyster counts declined substantially between 2018 and 2020, potentially biasing estimates of change based on absolute counts. Specifically, absolute abundance changes are likely underestimated for low-abundance clusters in 2018 (e.g., gNY) and overestimated for initially dominant clusters (e.g., gVA) since the total sample size collected from reefs as a whole decreases over time, regardless of genetic cluster membership. Frequency-based analyses, by accounting for total sample size per reef, better reflect changes in the relative survival of genetic clusters over time in the context of declining overall reef live densities.

Section S5. Concordance in the outcomes of Fisher's Exact Tests on the relationship between genetic cluster frequencies and sampling year across the 24 assignment sets

Fisher's Exact Tests indicated a significant association between genetic cluster and sampling year for most of the reefs on which gVA and gNY occurred, but not gME or gMA (Table S2). The number of reefs showing significant shifts was greater than expected by chance for gVA and gNY (permutation p-values: p=0.002 for both), but not for gME (permutation p-value, p=0.088) or gMA (permutation p-value, p =1.0). Notably, gNY consistently increased in frequency between 2018 and 2020, while gVA consistently decreased. These trends were observed across all 24 assignment datasets (Figure S9), although a subset of datasets also

identified significant shifts in gME, albeit on fewer reefs overall.

Section S6. Additional support for shifts in the genetic composition of reefs over time from distance-based methods

We analyzed reef compositional data using PERMANOVA to provide further context to the shifts in the relative prevalence of genetic clusters on reefs over time. We found that the composition of reefs was strongly dependent on sampling year, with a non-significant effect of block (PERMANOVA: year, p=0.001; block, p=0.116; Figure S10). Fall 2020 reefs were characterized by lower relative frequencies of groups gVA and gMA compared to reefs in 2018, and higher frequencies of gNY and gME. A test for multivariate dispersion via the betadisper function in the R vegan package confirmed this pattern between sampling years was not due to differences in within-year variability (dispersion) in reef-level genetic cluster frequency (p=0.387). Simper analyses with 1000 permutations indicated that the frequency of two genetic clusters contributed significantly to dissimilarity between the two sampling years: cluster gVA was more abundant on reef sampled in 2018 (Average abundance in 2018 = 0.442; average abundance in 2020 = 0.088; Bonferroni-adjusted p-value=0.004) while cluster gNY was more abundant in 2020 (Average proportional abundance in 2018 = 0.131; average proportional abundance in 2020 = 0.442; Bonferroni-adjusted p-value=0.016). Clusters gME and gMA did not contribute significantly to dissimilarity between sampling years (Bonferroni-adjusted p-values= 1.000 and 0.184, respectively).

Section S7. Discussion of results relating to within-cluster genetic diversity

The results of our analyses support a few consistent within-cluster genetic diversity patterns that may relate to the relative performance of genetic clusters on our mixed experimental reefs. Namely, estimates of genetic diversity for gNY, the genetic cluster that survived at a greater rate on reefs, were generally either higher within this group or changed differently within this group over time as compared to the other oyster genetic clusters. Estimates of H_e and A_r at the start and end of the experiment were significantly higher for oysters from gNY than all other clusters (Figure 1.4b,d). Observed heterozygosity (H_o) also tended to be higher for reef-level samples from this cluster at the start of the experiment (Figure 1.4a), although likelihood ratio tests supported dropping the term for genetic cluster identity from this model. Shifts over time in most reef-level genetic diversity metrics tended to be consistent among genetic clusters with the exception of F_{IS} and average within-cluster pairwise relatedness, which showed significant shifts for only gNY (Figure 1.4c). Pairwise relatedness decreased significantly between sampling years among individuals within gNY, but did not shift significantly within any of the other genetic clusters. Similarly, while reef-level F_{IS} did not change over time for genetic clusters gME, gMA, or gVA, it increased significantly for gNY such that the heterozygote excess detected in fall 2018 was no longer present in most reef-level samples of gNY oysters on reefs in fall 2020; an indication that the distribution of genetic variation within and among individuals surviving to fall 2020 had shifted over time in this cluster. While difficult to discern the exact mechanism for this redistribution of genetic diversity within gNY away from heterozygote excess, this pattern would be consistent with stronger structure emerging over time associated with distinct family groups (i.e., decreasing pairwise relatedness).

Section S8. Variation between genetic clusters in fall 2018 condition index not independently explained by either dry tissue or shell mass

Patterns in condition index were not clearly related to either dry tissue or shell mass alone. While we found either significant or marginally significant interactions between year and genetic cluster for dry shell weight (Monte Carlo permutation tests for dry shell mass, year * genetic cluster: $F_{df=3} = 6.981$, p=0.001; Table S17) and dry tissue weight (Monte Carlo permutation tests for dry shell mass, year * genetic cluster: $F_{df=3} = 2.593$, p=0.057; Table S18) respectively, neither trait differed significantly among genetic clusters in fall 2018. However, the mean condition index of genetic clusters on reefs sampled in 2018 showed a significant, moderate correlation between condition index and dry tissue mass (Spearman's rho $\rho = 0.488$, p-value =0.001), but not dry shell mass (Spearman's rho $\rho = 0.053$, p-value =0.743).

Statistically significant differences in both tissue mass and shell mass emerged over time: in 2020, gMA oysters had significantly greater shell mass than oysters from gME and gNY (Tukey post-hoc contrasts, $p<0.05$). Additionally, oysters from experimental block C tended to have greater dry tissue mass (Monte Carlo permutations; block: $F_{df=3} = 5.450$, p=0.007; Table S18) than oysters from blocks A and B, and greater shell mass than all other blocks (Monte Carlo permutations; Block: $F_{df=3} = 7.3937$, p=0.001; Table S17). However, differences in shell and tissue mass in Fall 2020 do not incorporate variation in shell dimensions (e.g. shell length and height) present at this time period (see Figure 1.5 and discussion in main text).

Supplementary Figures.

Figure S1. PCAs with 95% confidence ellipses corresponding to clusters for each of the 6 hatchery label-oyster grower combinations from analyses using (a) only the initial samples prior to reef construction (n=5605 filtered SNPs, 110 individuals; ANGSD-generated genotype likelihoods), versus incorporating (b) all samples from initial, 2018, and 2020 collections and visualizing only the subset of initial library samples (n= 4476 filtered SNPs from the full 803 individual dataset with only the 104 initial library individuals visualized; dDocent/FreeBayes-generated genotype calls). The PCA in panel (c) shows the realized assignments of individuals from initial samples to the 4 best genetic clusters for the primary assignment set used in main analyses projected onto the PCA in panel b, with groups colored according to the inferred hatchery source label to which it is likely to correspond.

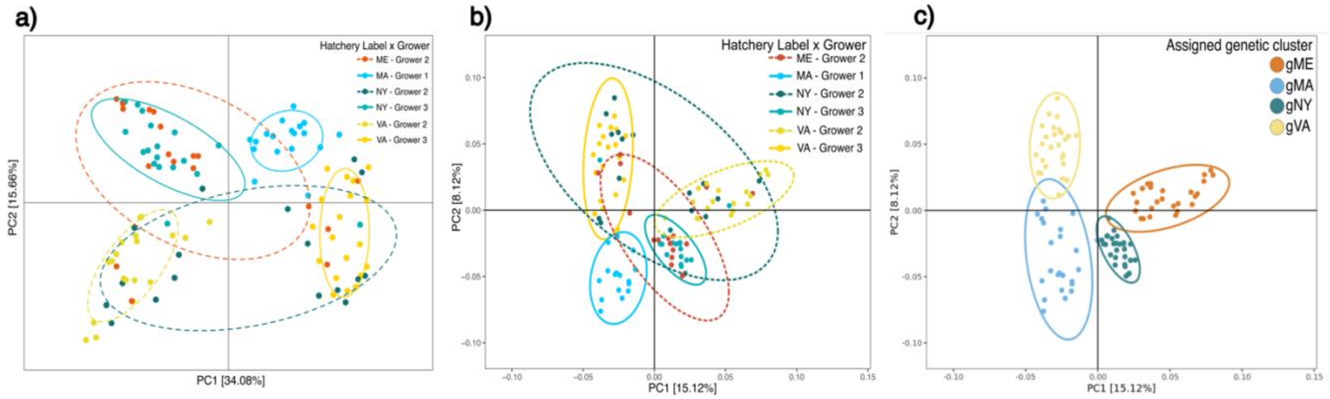

Figure S2. Missing data profiles and walk-through of filtering steps to treat batch-specific signatures in the frequency of missing data per individual. Plots depicting rates of individual missingness (proportion of missing genotypes for each individual out of total called genotypes/sites). Points represent individual samples. Source ddRAD library batch is indicated by color: green=2018 batch; orange = 2020 batch; purple = initial batch. Panel (a) shows the distinct batch library-specific signature for higher frequencies of missing data per individual for the fall 2020 batch apparent at the start of our SNP filtering. Pruning for individual missingness at the recommended dDocent threshold (red dashed line) would remove a large proportion of fall 2020 batch individuals, whereas our slightly modified threshold (blue dashed line) retains most of these individuals. Panel (b) shows rates of missing data per individual after applying this initial filtering step to address both overall and batch-specific signatures for missing data. With this SNP set, we then pruned individuals with a frequency over 16% missing genotypes as depicted in panel (c). An example of the resultant frequencies of missing data per individual following these filtering steps is shown in panel (d).

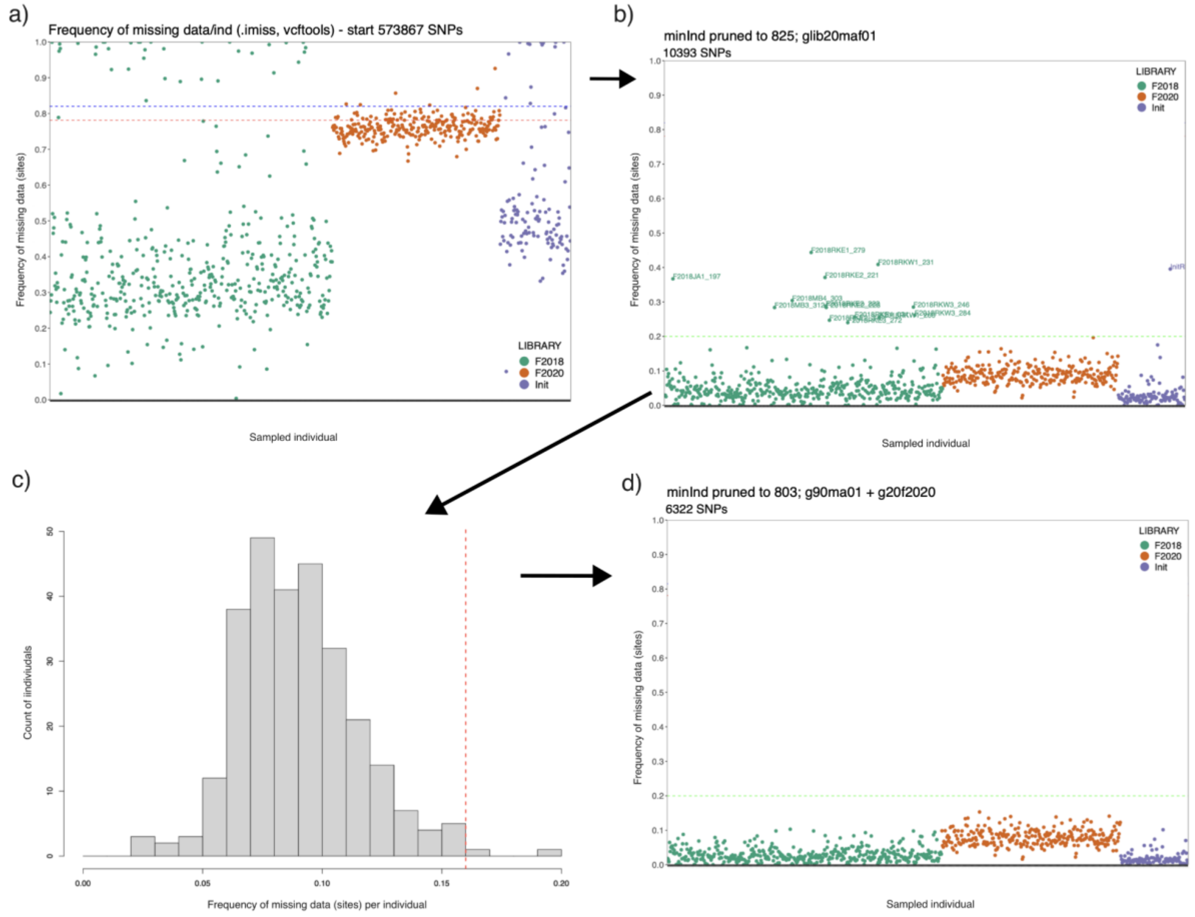

Figure S3. Diagnostic plots depicting (a) the distribution of Bayesian Information Criterion (BIC) values generated for K=1 through K=10 across 25 DAPC runs, and (b) absolute changes in BIC (K-statistic provided by DAPC output) between successive values of K and the (c) unstandardized and (d) standardized change in these ΔBIC values, as calculated in Evanno, Regnaut, and Goudet (2005). K=4 is identified as a threshold point in K values distinguishing larger changes in BIC among successive values of K.

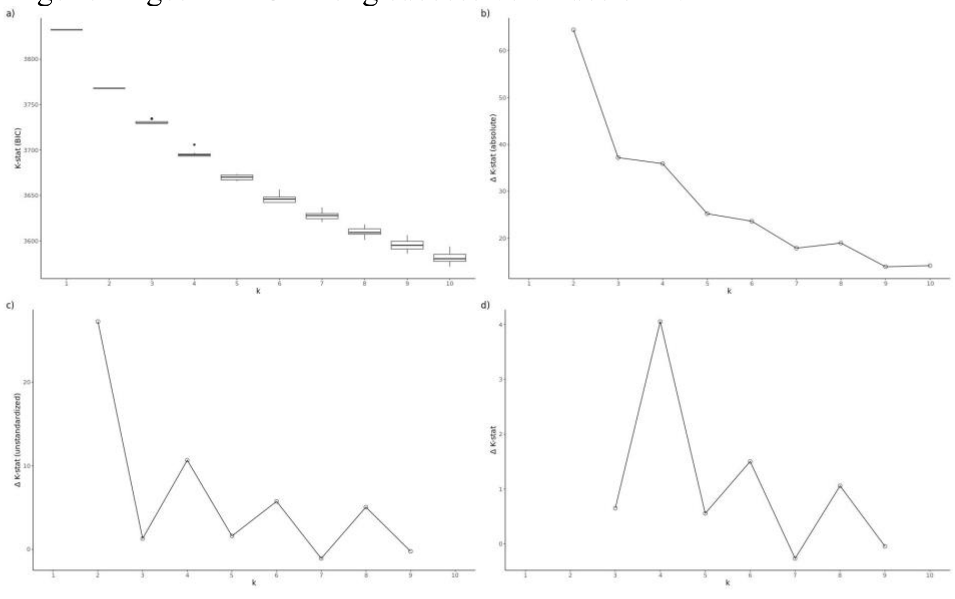

Figure S4. Diagnostic plots depicting (a) the distribution of sNMF-generated cross-entropy (CE) values for 100 replicate runs for each value of K=2 through K=10, the (b) absolute changes in CE between successive values of K and the (c) standardized change in these ΔCE values, as calculated in Evanno, Regnaut, and Goudet (2005). K=4 is identified as a threshold point in K values distinguishing larger changes in CE among successive values of K. Note, K values on x-axis begin at 2 because K=1 was not examined within our sNMF runs.

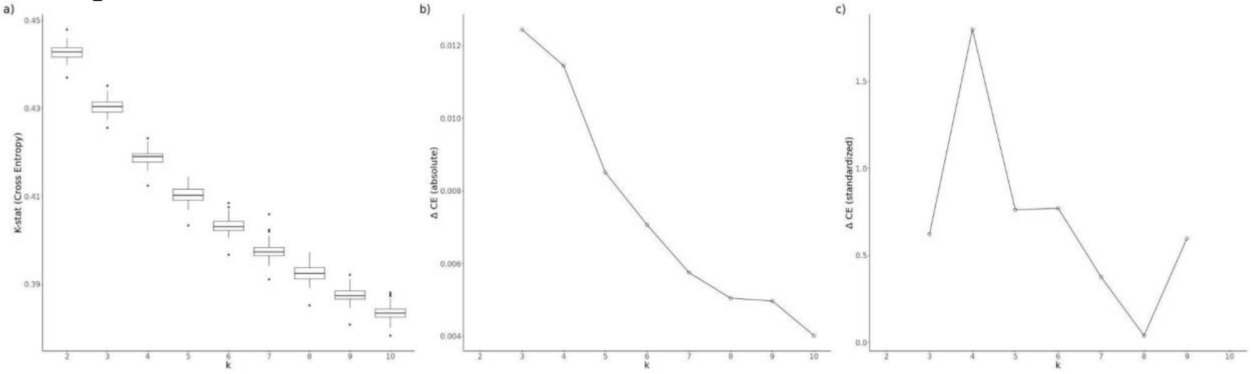

Figure S5. (a) Heatmap depicting pairwise comparisons of the proportion of assignments that are not concordant between the 12 SNP sets x DAPC vs. sNMF assignment approaches. Darker values indicated more concordant assignment pairs, while lighter values indicate pairs with more disagreements in assignments (maximum=0.166). (b) Mean and standard error of the proportion of discordant assignments between pairwise comparisons of each of the 24 assignment sets (overall average=7.75%, ± 0.2% SE). Individual assignments tended to be most similar within sets applying the same assignment approach, i.e., sNMF or DAPC, and this distinction represented the largest source of incongruence among assignment sets. Generally, SNP sets that were filtered for fall 2020 site missingness tended to return more similar assignments between DAPC and sNMF. The asterisks in both plots indicate the primary assignment set used for figures and analyses presented in the main text.

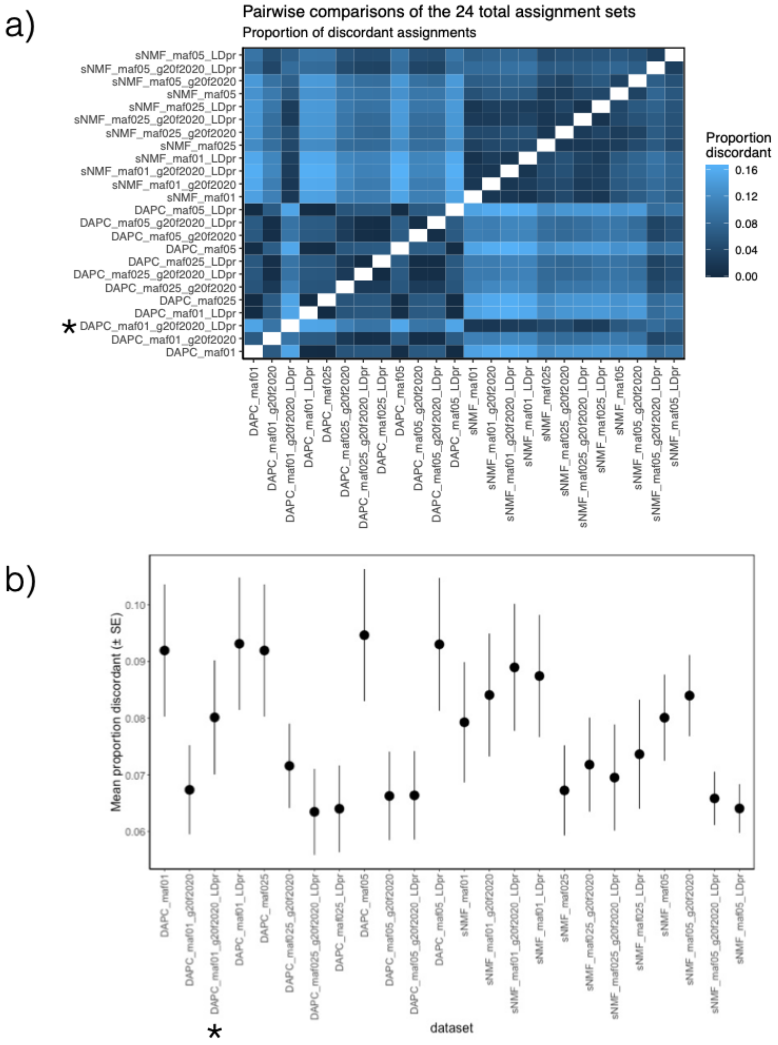

Figure S6. Concordance across all 24 assignment sets (12 SNP subsets × 2 assignment methods) for (a) average reef-level changes in genetic cluster frequencies between sampling years, and (b) the estimated effects of sampling year on each cluster based on marginal means contrasts.

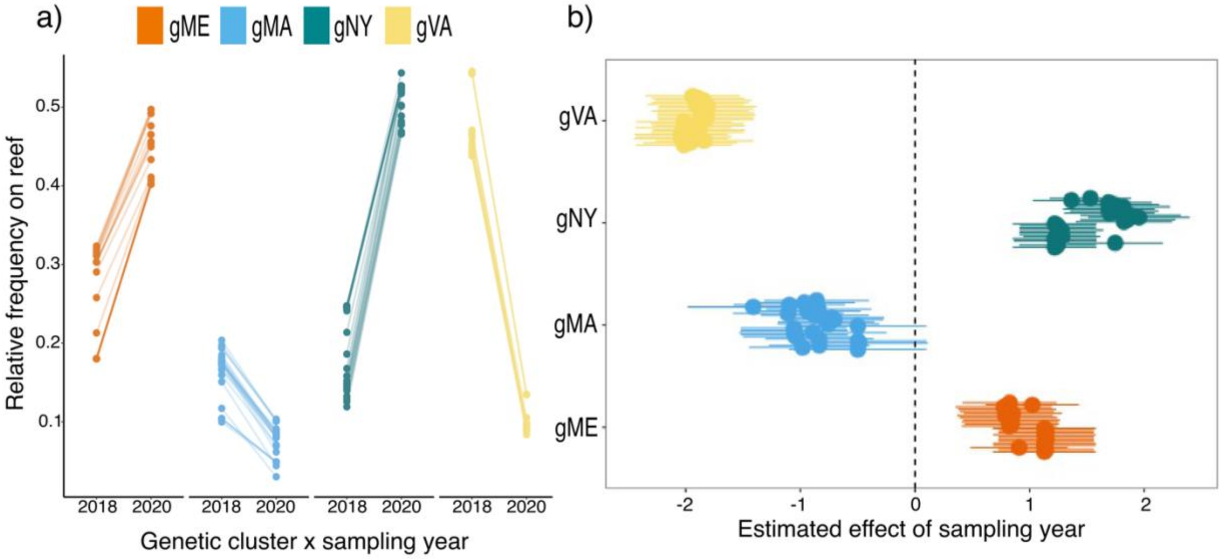

Figure S7. Change in reef-level absolute abundances (raw counts) of oysters from four genetic clusters across sampling years, based on the primary assignment set used in main analyses. Bold lines and points show reef-averaged means (± SE); faint lines and points indicate individual reef trajectories. Asterisks denote significant changes across years (*** P < 0.001, ** P < 0.01, * P < 0.05). In panels b and c, are stacked bar plots showing reef-level counts of individuals from each genetic cluster in (b) 2018 and (c) 2020. Each bar represents one reef; cluster identity is indicated by color: orange = gME, blue = gMA, green = gNY, yellow = gVA.

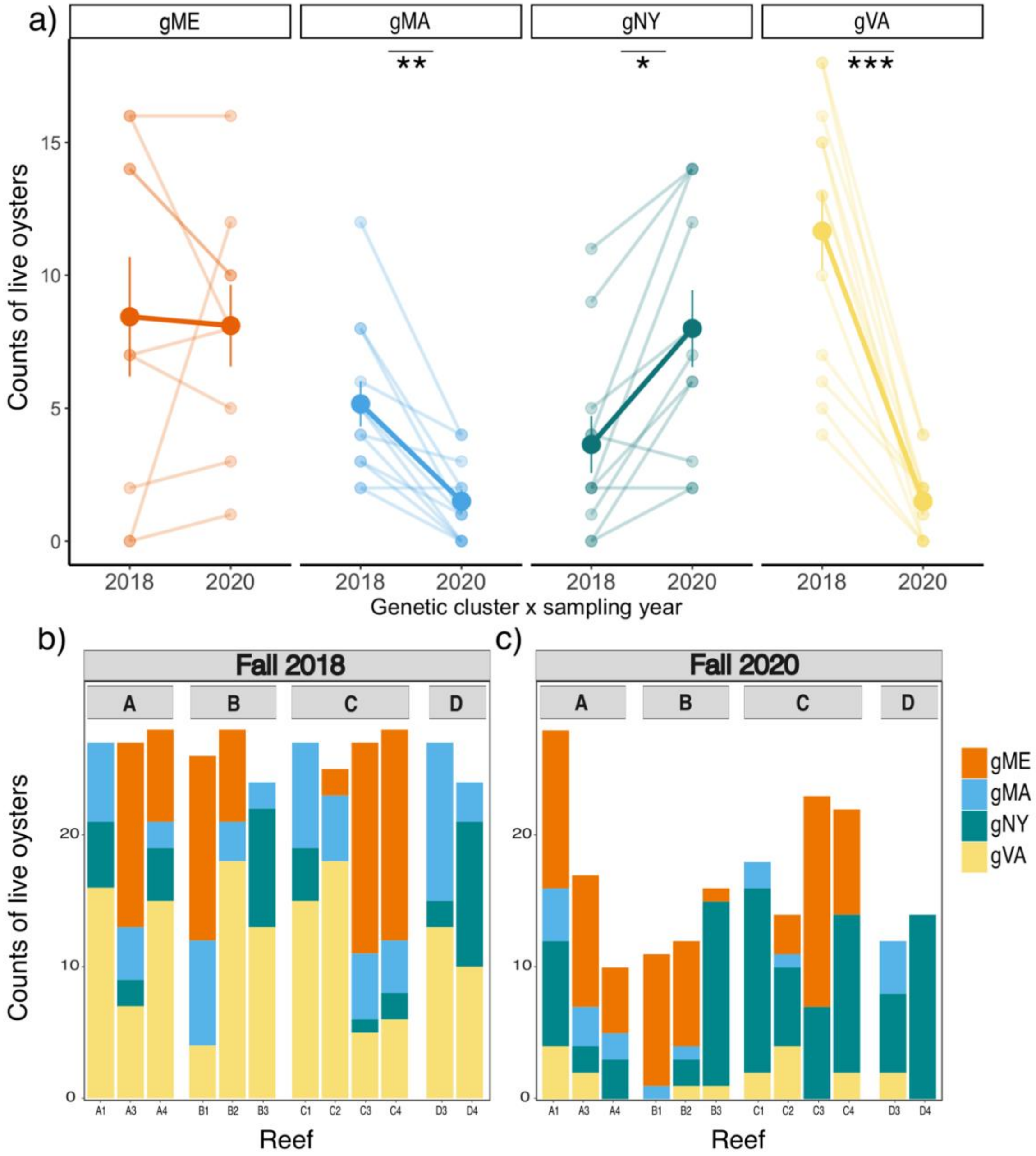

Figure S8. Concordance across all 24 assignment sets (12 SNP subsets × 2 assignment methods) in (a) average reef-level changes in absolute counts of oysters from each genetic cluster, and (b) estimated effects of sampling year on cluster-level counts based on marginal means contrasts.

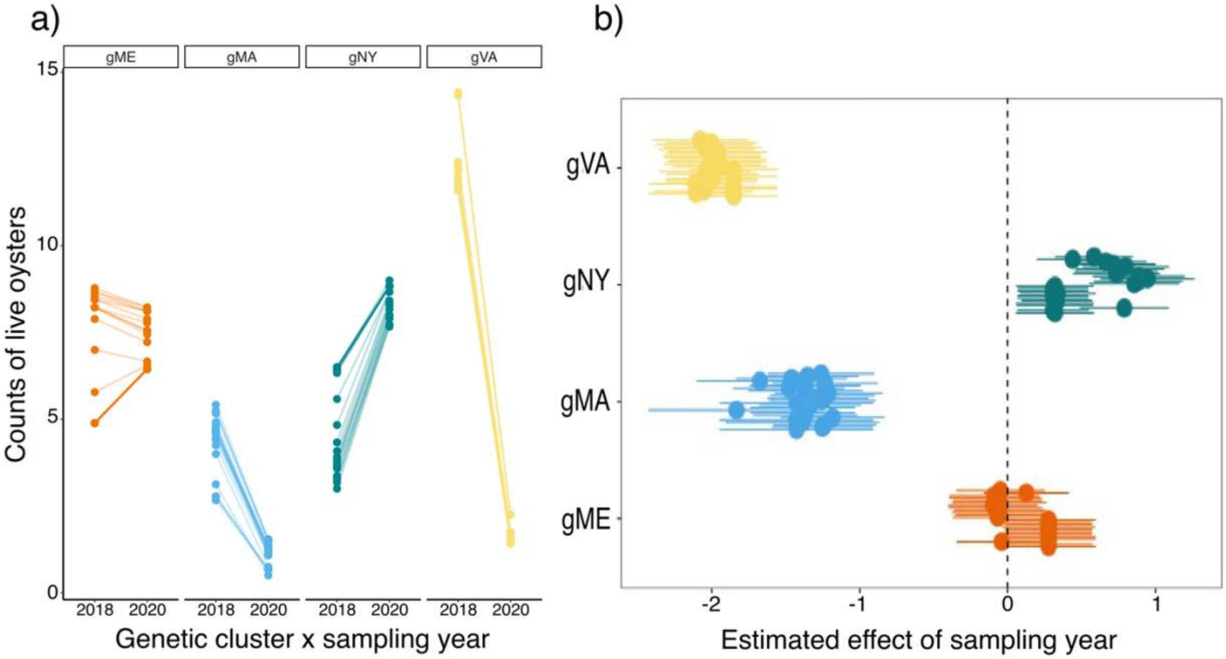

Figure S9. Results of Fisher's Exact Test and associated permutations evaluating changes in genetic cluster frequencies on reefs between 2018 and 2020 across the 24 genetic assignment sets. Genetic cluster identities (gME, gMA, gNY, gVA) are shown along the x-axis, and the genetic assignment datasets are listed on the y-axis. The counts of reefs (a) with significant shifts in the frequency of each genetic cluster based on Fisher's Exact Tests ($p < 0.05$). Cells are shaded in blue where the counts of significant reefs for a cluster was greater than that expected under the null distribution generated by permuting sampling year labels within each reef (1000 iterations). Permutation-derived p-values (b) for each genetic cluster and genetic assignment dataset, indicating whether the counts of reefs with significant shifts for a given genetic cluster (a) was greater than expected by chance.

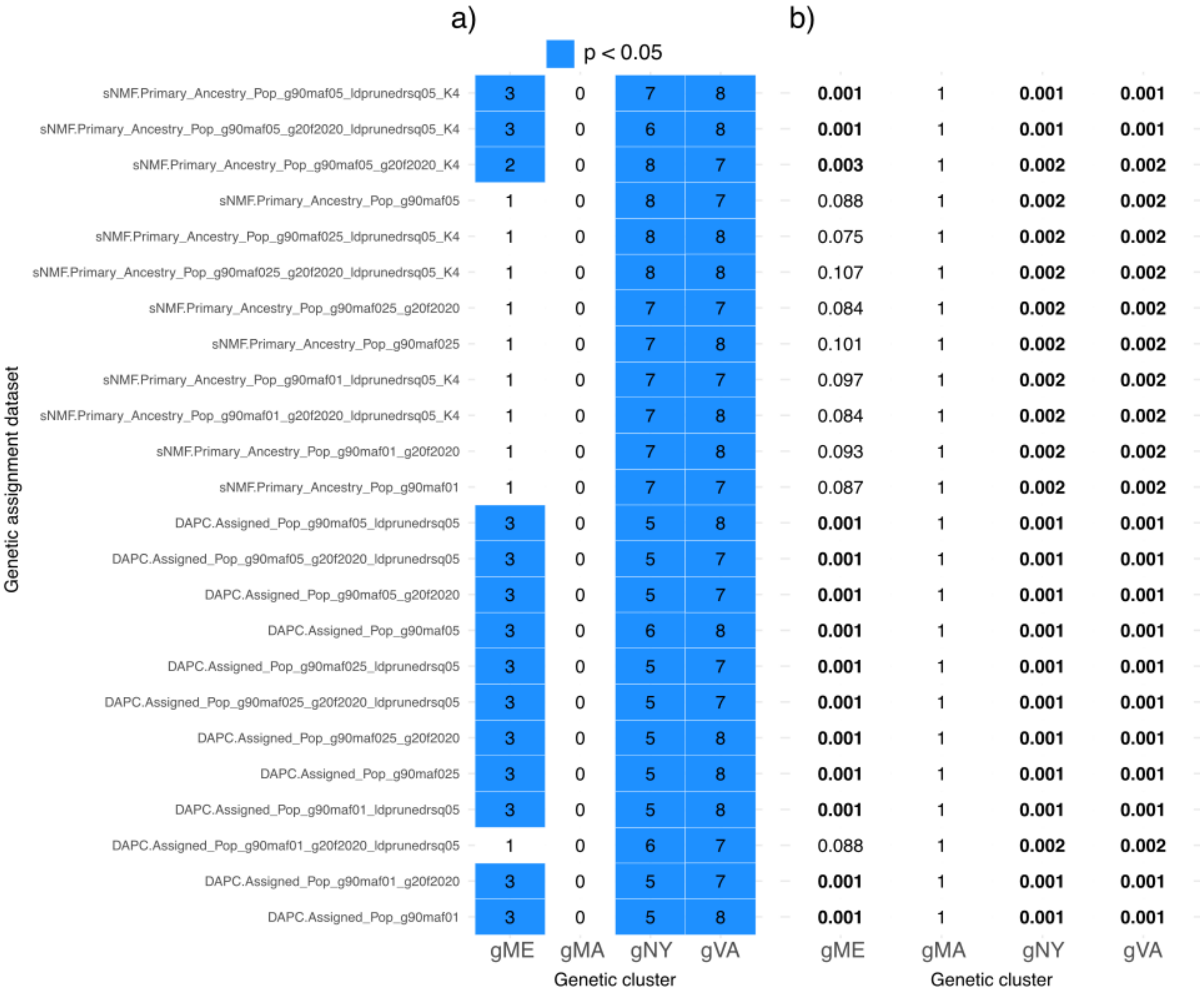

Figure S10. Non-metric multidimensional scaling (nMDS) plot depicting the composition of oyster reefs by the four genetic clusters in fall 2018 (black) and fall 2020 (light gray). Ellipses indicate the 95% confidence intervals for sampling year. Points represent the relative composition of the 12 oyster reefs sampled in 2018 and 2020, with reef trajectories depicted by gray dotted lines and arrows. Bolded arrows represent correlations between the nMDS ordination axes and the relative abundance patterns for each genetic cluster (gME, gMA, gNY, gVA).

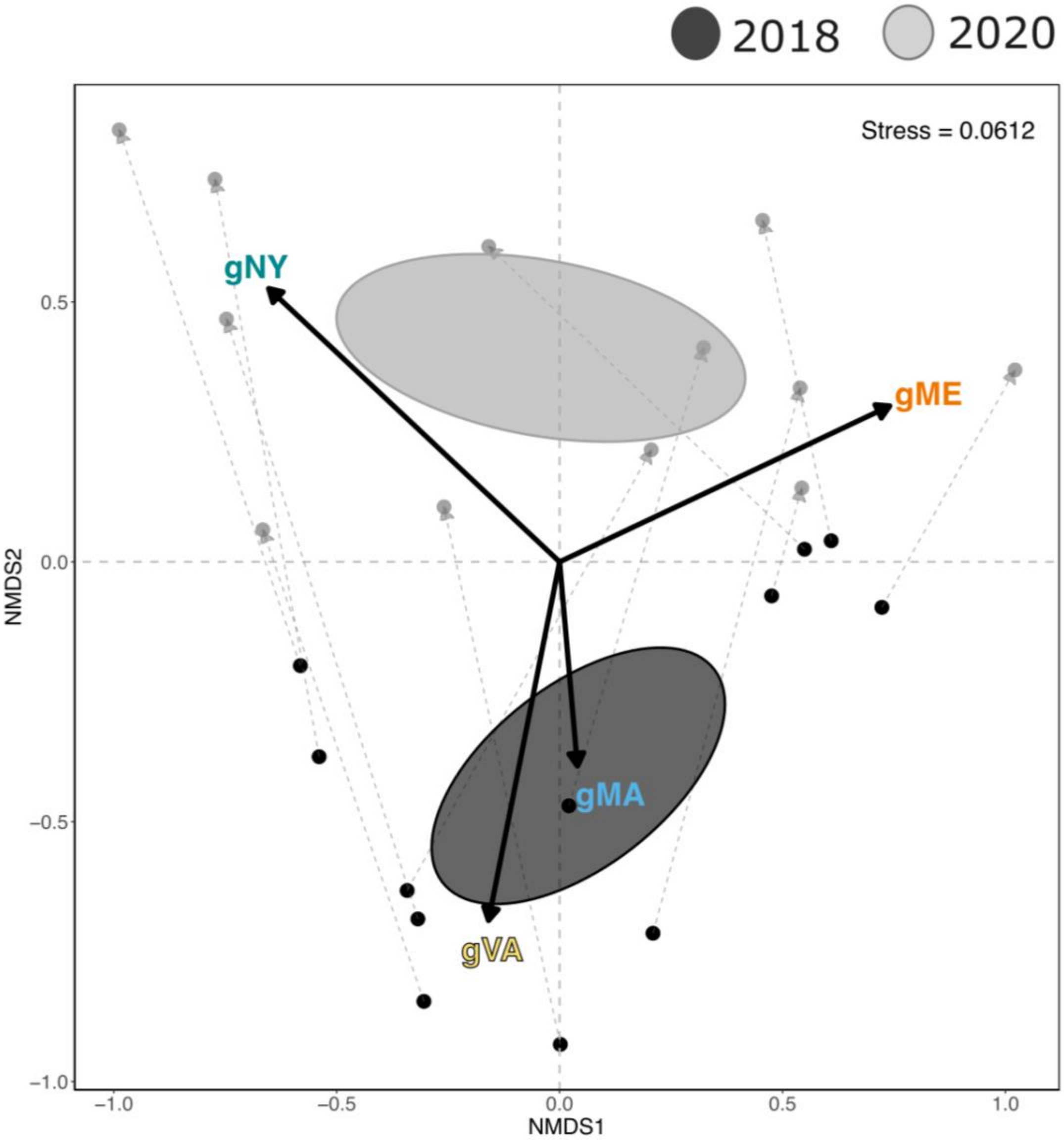

Figure S11. Standardized coefficients from the multiple regression of the relationship between the change in the reef-level frequency of genetic clusters and the average values of 8 oyster traits in fall 2018 using the (a) full dataset versus (c) the dataset in which we pruned the two high dfbetas values. Both models were fit to normalized (z-transformed) trait values to generate standardized estimates of the coefficients for all traits. Significance for the probability of the t-statistic of each coefficient is indicated by the following: *** $p < 0.001$, ** $p < 0.01$, * $p < 0.05$, . $p < 0.1$. The partial regression plots of the relationship between the change in the relative frequency of genetic clusters on individual reefs over time and the mean condition index measured in 2018 using the using the (b) full dataset versus (d) the dfbetas-pruned dataset.

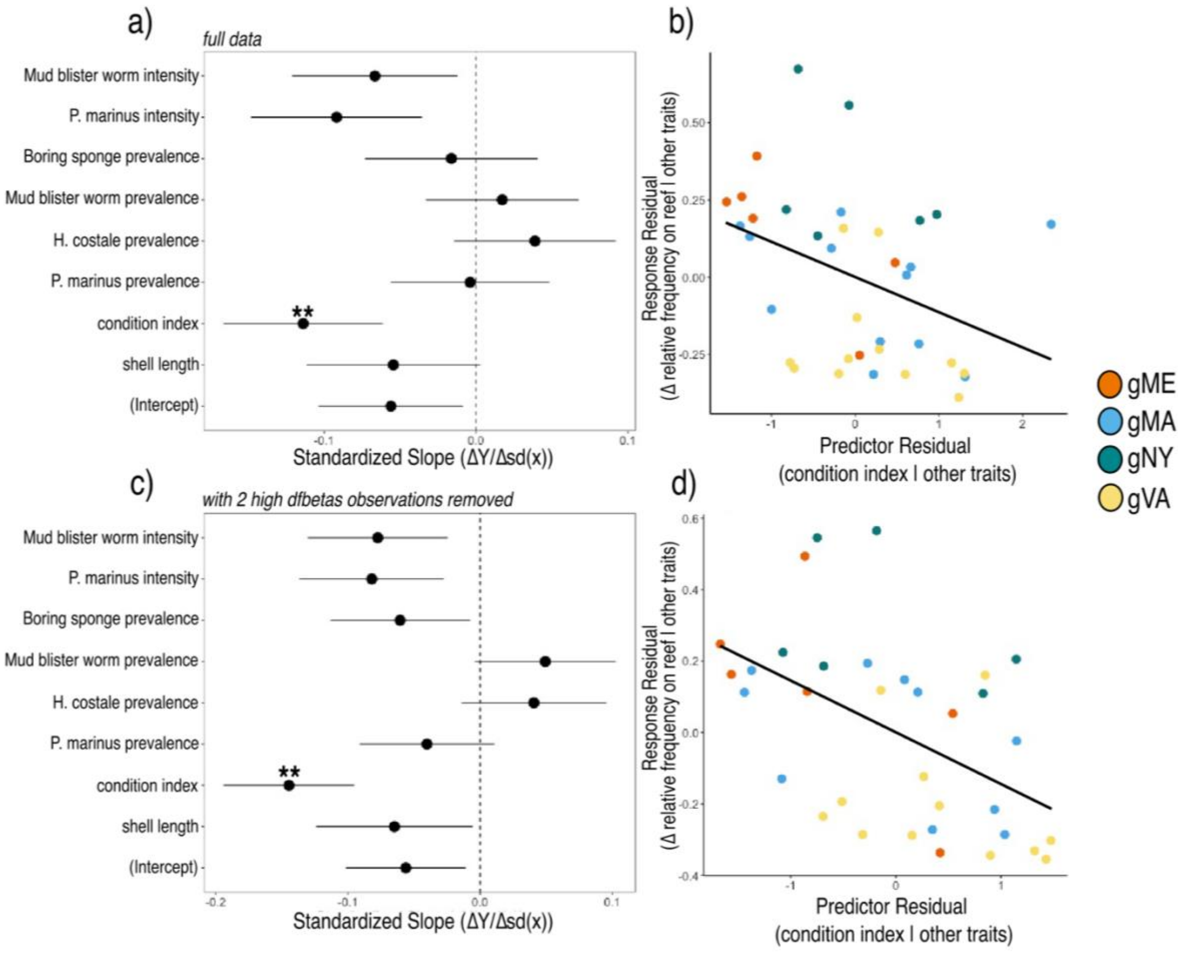

Figure S12. Pairwise F_{ST} estimates among the 4 genetic clusters assigned from initial samples (n=104 individuals) prior to the experiment show the greatest extent of differentiation between gME and gVA, and the least differentiation between gME and gNY, and gMA and gVA.

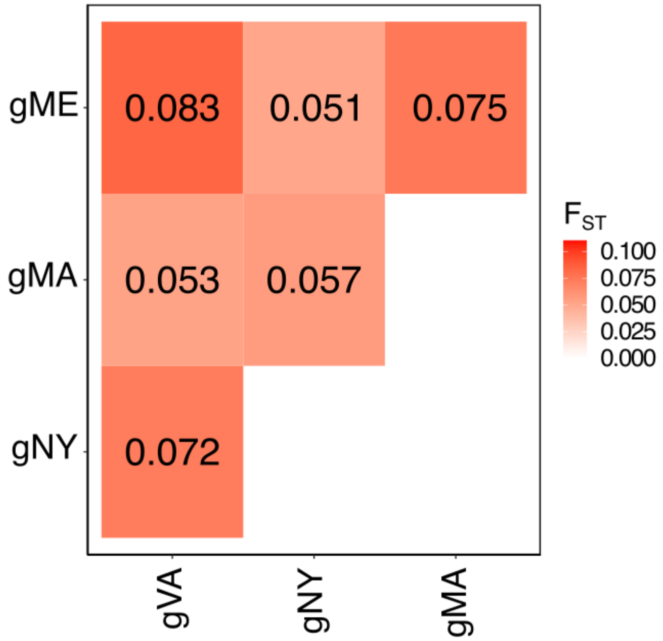

Figure S13. Plot depicting variation in mean individual pairwise relatedness within each genetic cluster (depicted by color) in fall 2018 and fall 2020. Reef-level estimates of mean individual pairwise relatedness, shown as fainter points and lines, were derived from a bootstrapping approach. Bold points show the mean estimates (± SE) for each genetic cluster. Letters indicate significant pairwise differences ($p<0.05$) identified with Tukey-Kramer post-hoc analyses.

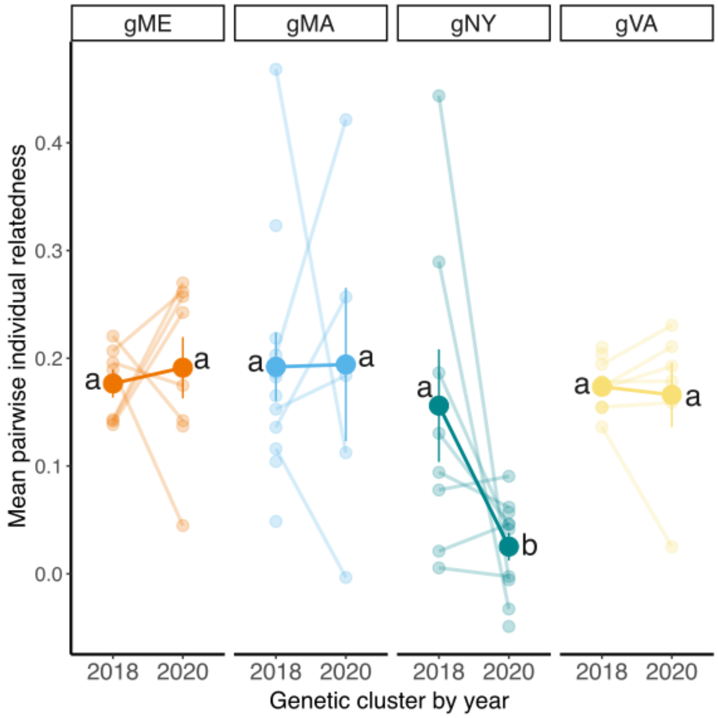

Figure S14. Plots depicting variation in parasite infection among oyster genetic clusters (gME, gMA, gNY, gVA) in fall 2018. Panels show patterns of parasite infection for each parasite species ordered by overall infection frequency, excluding outcomes for the most prevalent parasite *P. marinus* (presented in the main text): (a) mud blister worm (*Polydora sp.*) prevalence and (b) infection intensity; (c) boring sponge (*Cliona spp.*) prevalence; and (d) *H. costale* prevalence. Bold points represent genetic cluster means (± SE) averaged across reefs; smaller, semi-transparent points show reef-level values. Letters with an asterisk (*) indicate pairwise comparisons that are marginally significant ($0.05 \leq p \leq 0.1$) in Tukey-Kramer post-hoc analyses.

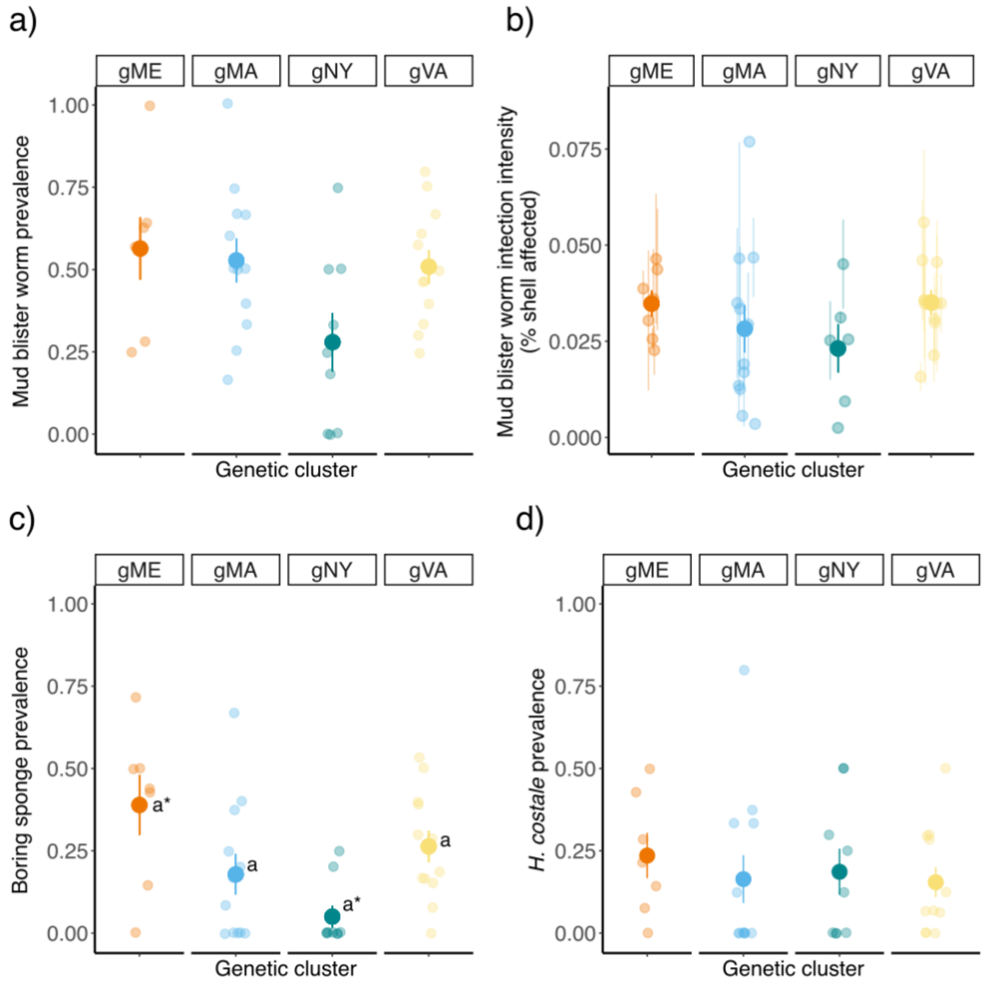

Supplement: Supplementary file 1 — Appendices S1‐S8 and Figures S1–S14. [file EVA-18-e70128-s001.pdf]
