# Supplementary material for: Non‐Random Mortality in an Experimental Oyster Restoration
Source: Evol Appl. 2025 Jul 6;18(7):e70128. doi: 10.1111/eva.70128 (PMC12229816; doi:10.1111/eva.70128)
Supplement: Supplementary file 2 — Tables S1–S18. [file EVA-18-e70128-s002.pdf]

Supplementary Tables: Truskey et al. Non-random mortality in an experimental oyster restoration. *Evolutionary Applications*

Table S1. Summary of SNP data filtering steps

Table S2. Fisher's Exact Test output on the relationship between genetic cluster frequencies and sampling year

Tables S3-7. Anova tables from GLMMs for within-cluster genetic diversity

Tables S8-14. Anova tables from LMM and GLMMs for oyster traits

Tables S15-16. Anova and marginal means contrast tables for GLMM fit with raw counts rather than frequencies

Tables S17-S18. Permutation test results for fixed effects in LMM of oyster dry shell weight and dry tissue weight, with reef as a random intercept

Table S1. Summary of SNP data filtering steps

Table S1. Summary of SNP data filtering steps. Rows represent filtering steps, while columns provide statistics at each step. 'Sites' refer to polymorphisms (indels and SNPs, with only bi-allelic SNPs retained in the final step), and 'Inds' denote the number of individual samples. "Start" and "End" columns show counts before and after applying each filter, while "Removed" columns indicate the number of sites or individuals excluded. Key counts are highlighted: the number of sites within the base filtered SNP set (*blue, italic text*), the total number of individuals passing missing data filters and used in the final SNP sets (green highlighted text), the total number of bi-allelic SNPs in each of the 12 final SNP sets (**bold** text), and the total number of bi-allelic SNPs in the primary SNP set used in analyses (**bold**, yellow highlighted text). The 12 SNP set names are as referenced in the main text.

SNP set name	Filter step	Filter details	Start sites	End sites	Removed sites	Start Inds	End Inds	Removed Inds
Base minimally filtered SNP set	1	Minor allele count > 3, Mean site quality > 20, Site call rate > 0.5, Minimum depth > 3	3434126	*573867*	2860259	885	885	0
	2	Filter_missing_ind script: Ind call rate cutoff, 0.815	573867	573867	0	885	825	60
	3	Rate of genotype missingness per library < 0.2; Minor allele frequency (MAF) > 0.01	573867	10398	563469	825	825	0
base 803 inds filtered for missing data	4	Filter_missing_ind script: Ind call rate cutoff, 0.16	10398	10398	0	825	803	22
	1	Site call rate > 0.90; MAF > **0.01**	*573867*	11680	562187	803	803	-
	2	dDocent_filters script	11680	7447	4233	803	803	-
	3	Decomposed to allelic primitives + removed indels	9061	8518	543	803	803	-
SNP set 1	4	Max alleles=2	8518	**8240**	278	803	803	-
SNP set 7	5	LD-pruning in PLINK with r^2=0.5	8240	**6029**	2211	803	803	-
	1	Site call rate > 0.90;	*573867*	11680	562187	803	803	-

		MAF > 0.01						
	2	Remove loci missing in more than 20% of Fall 2020 inds	11680	9149	2531	803	803	-
	3	dDocent_filters script	9149	5684	3465	803	803	-
	4	Decomposed to allelic primitives + removed indels	6965	6537	428	803	803	-
SNP set 4	5	Max alleles=2	6537	**6322**	215	803	803	-
SNP set 10	6	LD-pruning in PLINK with r^2=0.5	6322	**4679**	1643	803	803	-
	1	Site call rate > 0.90; MAF > 0.025	*573867*	7789	566078	803	803	-
	2	dDocent_filters script	7789	5353	2436	803	803	-
	3	Decomposed to allelic primitives + removed indels	6194	5912	282	803	803	-
SNP set 2	4	Max alleles=2	5912	**5767**	145	803	803	-
SNP set 8	5	LD-pruning in PLINK with r^2=0.5	5767	**4327**	1440	803	803	-
	1	Site call rate > 0.90; MAF > 0.025	*573867*	7789	566078	803	803	-
	2	Remove loci missing in more than 20% of Fall 2020 inds	7789	6075	1714	803	803	-
	3	dDocent_filters script	6075	4065	2010	803	803	-
	4	Decomposed to allelic primitives + removed indels	4716	4504	212	803	803	-
SNP set 5	5	Max alleles=2	4504	**4393**	111	803	803	-
SNP set 11	6	LD-pruning in PLINK with r^2=0.5	4393	**3371**	1022	803	803	-
	1	Site call rate > 0.90; MAF > 0.05	*573867*	5211	568656	803	803	-
	2	dDocent_filters script	5211	3729	1482	803	803	-

	3	Decomposed to allelic primitives + removed indels	4200	4020	180	803	803	-
SNP set 3	4	Max alleles=2	4020	**3946**	74	803	803	-
SNP set 9	5	LD-pruning in PLINK with r^2=0.5	3946	**3063**	883	803	803	-
	1	Mean site call rate > 0.90; MAF > 0.05	*573867*	5211	568656	803	803	-
	2	Remove loci missing in more than 20% of Fall 2020 inds	5211	4057	1154	803	803	-
	3	dDocent_filters script	4057	2794	1263	803	803	-
	4	Decomposed to allelic primitives + removed indels	3145	3019	126	803	803	-
SNP set 6	5	Max alleles=2	3019	**2960**	59	803	803	-
SNP set 12	6	LD-pruning in PLINK with r^2=0.5	2960	**2339**	621	803	803	-

Table S2. Fisher's Exact Test output on the relationship between genetic cluster frequencies and sampling year

Table S2. Outcome of Fisher's Exact Test to determine the association between individual genetic cluster membership (versus membership to others) and the sampling year. P-value adjustments for multiple testing were applied using the Benjamini-Hochberg method. Values in bold are significant (<0.05), while values in italic are marginally significant (0.05 < p < 0.1). Individual genetic cluster assignments used in this analysis are from the primary assignment set described in the main text.

Reef	Genetic cluster	Adjusted p-value	Direction of change
A1	**gME**	**0.0024**	**INC**
	gMA	0.6037	DEC
	gNY	0.6113	INC
	**gVA**	**0.0067**	**DEC**
A3	gME	0.7962	INC
	gMA	1	INC
	gNY	0.6971	INC
	gVA	0.5595	DEC
A4	gME	0.413	INC
	gMA	0.4448	INC
	**gNY**	0.4983	INC
	**gVA**	**0.0125**	**DEC**
B1	gME	0.1328	INC
	gMA	0.413	DEC
	gVA	0.4496	DEC
B2	gME	*0.0817*	*INC*
	gMA	1	DEC
	gNY	0.1773	INC
	**gVA**	**0.0097**	**DEC**
B3	gME	0.5333	INC

	gMA	0.6037	DEC
	**gNY**	**0.0125**	**INC**
	**gVA**	**0.0121**	**DEC**
C1	gMA	0.4448	DEC
	**gNY**	**0.0016**	**INC**
	**gVA**	**0.0165**	**DEC**
C2	gME	0.4824	INC
	gMA	0.5333	DEC
	**gNY**	**0.0067**	**INC**
	gVA	*0.051* ·	*DEC* ·
C3	gME	0.6304	INC
	gMA	0.132	DEC
	gNY	*0.051* ·	*INC* ·
	gVA	0.132	DEC
C4	gME	0.3182	DEC
	gMA	0.2413	DEC
	**gNY**	**0.0047**	**INC**
	gVA	0.5595	DEC
D3	gMA	0.7793	DEC
	gNY	0.0203	INC
	gVA	0.1773	DEC
D4	gMA	0.4448	DEC
	**gNY**	**0.0067**	**INC**
	**gVA**	**0.0203**	**DEC**

Supplementary Tables S3-7. Anova tables from GLMMs for within-cluster genetic diversity

Table S3. Results from a Type II Sums of Squares Analysis of Deviance for **observed heterozygosity** on the main effect of sampling year. The GLMM was fit under a beta distribution with a logit link and a random intercept for experimental reef.

Explanatory variable	Chisq	Df	p-value
Year	63.338	1	< 0.0001***

Significance codes for probability of the test statistic under the null hypothesis: *** $p < 0.001$, ** $p < 0.01$, * $p < 0.05$, · $p < 0.1$; Chisq = chi-square test statistic, Df = degrees of freedom.

Table S4. Results from a Type II Sums of Squares Analysis of Deviance for **expected heterozygosity** on the main effects of sampling year and genetic cluster identity. The GLMM was fit under a beta distribution with a logit link and a random intercept for experimental reef.

Explanatory variable	Chisq	Df	p-value
Year	15.307	1	0.0001 ***
Genetic cluster	25.737	3	< 0.0001***

Significance codes for probability of the test statistic under the null hypothesis: *** $p < 0.001$, ** $p < 0.01$, * $p < 0.05$, · $p < 0.1$; Chisq = chi-square test statistic, Df = degrees of freedom.

Table S5. Results from a Type II Sums of Squares Analysis of Deviance for **allelic richness** on the main effects of sampling year and genetic cluster identity. The GLMM was fit under a beta distribution with a logit link and a random intercept for experimental reef.

Explanatory variable	Chisq	Df	p-value
Year	14.099	1	0.0002 ***
Genetic cluster	19.520	3	0.0002 ***

Significance codes for probability of the test statistic under the null hypothesis: *** $p < 0.001$, ** $p < 0.01$, * $p < 0.05$, · $p < 0.1$; Chisq = chi-square test statistic, Df = degrees of freedom.

Table S6. Results from a Type III Sums of Squares Analysis of Deviance for $\mathbf{F_{IS}}$ on the main effects of sampling year, genetic cluster identity, and their interaction. The GLMM was fit under a beta distribution with a logit link and a random intercept for experimental reef.

Explanatory variable	Chisq	Df	p-value
Intercept	18.284	1	< 0.0001 ***
Year	1.029	1	0.3103
Genetic cluster	1.390	3	0.7084
Year x Genetic cluster	15.724	3	0.0013**

Significance codes for probability of the test statistic under the null hypothesis: *** $p < 0.001$, ** $p < 0.01$, * $p < 0.05$, . $p < 0.1$; Chisq = chi-square test statistic, Df = degrees of freedom.

Table S7. Results from a Type III Sums of Squares Analysis of Deviance for **mean individual pairwise relatedness** on the main effects of sampling year, genetic cluster identity, and their interaction. Estimates of mean individual pairwise relatedness were derived from a bootstrapping approach to estimate mean relatedness values within individuals assigned to the same genetic cluster on each reef within a sampling year. The GLMM was fit under a beta distribution with a logit link and a random intercept for experimental reef.

Explanatory variable	Chisq	Df	p-value
Intercept	42.2394	1	< 0.0001 ***
Year	0.0116	1	0.9144
Genetic cluster	2.0250	3	0.5672
Year x Genetic cluster	10.1807	3	0.0171*

Significance codes for probability of the test statistic under the null hypothesis: *** $p < 0.001$, ** $p < 0.01$, * $p < 0.05$, . $p < 0.1$; Chisq = chi-square test statistic, Df = degrees of freedom.

Supplementary Tables S8-14. Anova tables from LMM and GLMMs for oyster traits

Table S8. Results of a Type III ANOVA via Satterthwaite's degrees of freedom method for square root-transformed oyster **shell height** and the main effects of sampling year, genetic cluster identity, and their interaction. Experimental reef was fit as a random intercept in this mixed effects model.

Explanatory variable	SS	MS	Df	DenDF	F value	p-value
Year	193.312	193.312	1	500.63	191.5392	< 2e-16 ***
Genetic cluster	15.769	5.256	3	483.40	5.2081	0.00151 **
Year x Genetic cluster	8.265	2.755	3	498.32	2.7298	0.04338 *

Significance codes for probability of the test statistic under the null hypothesis: *** p < 0.001, ** p < 0.01, * p < 0.05, . p < 0.1; SS = sums of squares, MS = mean square, DF = degrees of freedom, DenDF= denominator degrees of freedom via Satterthwaite's degrees of freedom method; F value = F statistic.

Table S9. Results of a Type III ANOVA via Satterthwaite's degrees of freedom method for square root-transformed **shell length** and the main effects of sampling year, genetic cluster identity, their interaction, and experimental block. Experimental reef was fit as a random intercept in this mixed effects model.

Explanatory variable	SS	MS	Df	DenDF	F value	p-value
Year	106.626	106.626	1	503.06	218.2066	< 2e-16 ***
Genetic cluster	20.471	6.824	3	411.36	13.9647	1.061e-08***
Block	3.800	1.267	3	8.77	2.5920	0.1190
Year x Genetic cluster	7.154	2.385	3	500.23	4.8803	0.0024**

Significance codes for probability of the test statistic under the null hypothesis: *** p < 0.001, ** p < 0.01, * p < 0.05, . p < 0.1; SS = sums of squares, MS = mean square, DF = degrees of freedom, DenDF= denominator degrees of freedom via Satterthwaite's degrees of freedom method; F value = F statistic.

Table S10. Results of a Type III ANOVA via Satterthwaite's degrees of freedom method for square root-transformed oyster **condition index** and the main effects of sampling year, genetic cluster identity, and their interaction. The LMM was fit with a random intercept for experimental reef.

**Explanatory variable**	**SS**	**MS**	**Df**	**DenDF**	**F value**	**p-value**
Year	1.90091	1.90091	1	475	33.0341	1.621e-08 ***
Genetic cluster	0.24028	0.08009	3	475	1.3919	0.24452
Year x Genetic cluster	0.43763	0.14588	3	475	2.5351	0.05621 .

Significance codes for probability of the test statistic under the null hypothesis: *** $p < 0.001$, ** $p < 0.01$, * $p < 0.05$, . $p < 0.1$; SS = sums of squares, MS = mean square, DF = degrees of freedom, DenDF= denominator degrees of freedom via Satterthwaite's degrees of freedom method, F value = F statistic.

Table S11. Results from a Type II Sums of Squares Analysis of Deviance for **the presence of infections by the microparasite *P. marinus*** on the main effects genetic cluster identity and experimental block. The GLMM was fit under a binomial distribution with a logit link and a random intercept for experimental reef.

Explanatory variable	Chisq	Df	p-value
Genetic cluster	26.692	3	6.83e-06 ***
Block	19.613	3	0.0002 ***

Significance codes for probability of the test statistic under the null hypothesis: *** p < 0.001, ** p < 0.01, * p < 0.05, · p < 0.1; Chisq = chi-square test statistic, Df = degrees of freedom.

Table S12. Results from a Type II Sums of Squares Analysis of Deviance for **the presence of infections by boring sponge** on the main effects of genetic cluster identity and experimental block. The GLMM was fit under a binomial distribution with a logit link and a random intercept for experimental reef.

Explanatory variable	Chisq	Df	p-value
Genetic cluster	6.6539	3	0.08379 ·

Significance codes for probability of the test statistic under the null hypothesis: *** p < 0.001, ** p < 0.01, * p < 0.05, · p < 0.1; Chisq = chi-square test statistic, Df = degrees of freedom.

Table S13. Results from a Type II Sums of Squares Analysis of Deviance for **the presence of infections by mud blister worms** on the main effect of experimental block. The GLMM was fit under a binomial distribution with a logit link and a random intercept for experimental reef.

Explanatory variable	Chisq	Df	p-value
Block	15.684	3	0.0013**

Significance codes for probability of the test statistic under the null hypothesis: *** $p < 0.001$, ** $p < 0.01$, * $p < 0.05$, · $p < 0.1$; Chisq = chi-square test statistic, Df = degrees of freedom.

Table S14. Results of permutation tests (n=1000 permutations) to evaluate the fixed effects of the LMM with the main effects of genetic cluster identity and experimental block on the **log-transformed intensity of infections by the *P. marinus* microparasite.** The LMM was fit with a random intercept for experimental reef that is preserved in permutations

**Explanatory variable**	**F value**	**Df**	**Df_{res}**	**p-value**	**permutation p-value**
Genetic cluster	3.893	3	179.119	0.010*	0.007**
Block	3.177	3	7.479	0.089 ·	0.031*

Significance codes for probability of the test statistic under the null hypothesis: *** $p < 0.001$, ** $p < 0.01$, * $p < 0.05$, . $p < 0.1$; F value = observed parametric test statistic (F-statistic) from the original model, Df = degrees of freedom, Df_{res}= residual degrees of freedom via Kenward-Roger method, p-value = parametric p-value, permutation p-value = p-value calculate using the permutation test as the proportion of permuted datasets in which the test statistic for the fixed effect is equal to or greater than the observed test statistic

Supplementary Tables S15-S16. Anova and marginal means contrast tables for GLMM fit with raw counts rather than frequencies (GLMMs)

Table S15. Results from a Type III Sums of Squares Analysis of Deviance for the observed **counts** of individuals present on reefs and the main effects of year, genetic cluster identity, and their interaction. The GLMM was fit under a negative binomial error distribution and a random intercept for experimental reef.

Explanatory variable	Chisq	Df	p-value
Intercept	97.9373	1	< 0.0001***
Year	0.0173	1	0.8952
Genetic cluster	19.3283	3	0.0002***
Year x Genetic cluster	46.3313	3	< 0.0001***

Table S16. Results from estimated marginal means contrasts of the effect of year on each genetic cluster from the model fit with counts of live oysters (gME-gVA). Results are averaged over the levels of the nested reef term and represented in the log odds ratio scale. P-values are adjusted for multiple testing using the Bonferroni method.

**Contrast**	**Genetic cluster**	**Estimate**	**SE**	**z-ratio**	**p-value**
2020 - 2018	gME	-0.0403	0.306	-0.132	1.000
2020 - 2018	gMA	-1.2368	0.349	-3.546	0.0016**
2020 - 2018	gNY	0.7885	0.301	2.615	0.0357*
2020 - 2018	gVA	-2.0513	0.336	-6.111	<0.0001***

Significance codes for probability of the test statistic under the null hypothesis: *** p < 0.001, ** p < 0.01, * p < 0.05, · p < 0.1

Supplementary Tables S17-S18. Permutation test results for fixed effects in LMM of oyster dry shell weight and dry tissue weight, with reef as a random intercept.

Table S17. Results of permutation tests (n=1000 permutations) to evaluate the fixed effects of the LMM with the main effects of sampling year, genetic cluster identity, and their interaction, and experimental block on the response variable **oyster dry shell weight.** The LMM was fit with a random intercept for experimental reef that is preserved in permutations.

**Explanatory variable**	**F value**	**Df**	**Df_{res}**	**p-value**	**permutation p-value**
Intercept	6.9698	1	21.6960	0.0151*	
Year	79.2623	1	471.9295	< 0.001***	0.001**
Genetic cluster	0.6279	3	424.4418	0.5973	0.581
Block	7.3937	3	8.0815	0.0106*	0.001**
Year x Genetic cluster	6.9814	3	468.4206	0.0001***	0.001**

Significance codes for probability of the test statistic under the null hypothesis: *** p < 0.001, ** p < 0.01, * p < 0.05, . p < 0.1; F value = observed parametric test statistic (F-statistic) from the original model, Df = degrees of freedom, Df_{res}= residual degrees of freedom via Kenward-Roger method, p-value = parametric p-value, permutation p-value = p-value calculate using the permutation test as the proportion of permuted datasets in which the test statistic for the fixed effect is equal to or greater than the observed test statistic

Table S18. Results of permutation tests (n=1000 permutations) to evaluate the fixed effects of the LMM with the main effects of sampling year, genetic cluster identity, and their interaction, and experimental block on the response variable **oyster dry tissue weight.** The LMM was fit with a random intercept for experimental reef that is preserved in permutations.

**Explanatory variable**	**F value**	**Df**	**Df_{res}**	**p-value**	**permutation p-value**
Intercept	7.5051	1	19.1738	0.013*	
Year	54.2102	1	471.8299	< 0.001***	0.001**
Genetic cluster	1.6101	3	443.7567	0.1863	0.174
Block	5.4495	3	8.0696	0.0243*	0.007**
Year x Genetic cluster	2.5928	3	467.7101	0.0521 ·	0.057 ·

Significance codes for probability of the test statistic under the null hypothesis: *** p < 0.001, ** p < 0.01, * p < 0.05, . p < 0.1; F value = observed parametric test statistic (F-statistic) from the original model, Df = degrees of freedom, Df_{res}= residual degrees of freedom via Kenward-Roger method, p-value = parametric p-value, permutation p-value = p-value calculate using the permutation test as the proportion of permuted datasets in which the test statistic for the fixed effect is equal to or greater than the observed test statistic
